# Protein Domain Guided Screen for Sequence Specific and Phosphorothioate-Dependent Restriction Endonucleases

**DOI:** 10.3389/fmicb.2020.01960

**Published:** 2020-08-18

**Authors:** Thomas Lutz, Honorata Czapinska, Alexey Fomenkov, Vladimir Potapov, Daniel F. Heiter, Bo Cao, Peter Dedon, Matthias Bochtler, Shuang-yong Xu

**Affiliations:** ^1^New England Biolabs, Inc., Ipswich, MA, United States; ^2^International Institute of Molecular and Cell Biology, Warsaw, Poland; ^3^Department of Biological Engineering, Massachusetts Institute of Technology, Cambridge, MA, United States; ^4^College of Life Science, Qufu Normal University, Qufu, China; ^5^Institute of Biochemistry and Biophysics PAS, Warsaw, Poland

**Keywords:** DNA backbone phosphorothioate modification, PT modification dependent endonucleases, SBD and HNH domain fusion, EcoWI endonuclease, Bsp305I endonuclease

## Abstract

Modification dependent restriction endonucleases (MDREs) restrict modified DNA, typically with limited sequence specificity (∼2–4 bp). Here, we focus on MDREs that have an SRA and/or SBD (sulfur binding domain) fused to an HNH endonuclease domain, cleaving cytosine modified or phosphorothioated (PT) DNA. We independently characterized the SBD-SRA-HNH endonuclease ScoMcrA, which preferentially cleaves 5hmC modified DNA. We report five SBD-HNH endonucleases, all recognizing GpsAAC/GpsTTC sequence and cleaving outside with a single nucleotide 3′ stagger: EcoWI (N7/N6), Ksp11411I (N5/N4), Bsp305I (N6/N4-5), Mae9806I [N(8-10)/N(8-9)], and Sau43800I [N(8-9)/N(7-8)]. EcoWI and Bsp305I are more specific for PT modified DNA in Mg^2+^ buffer, and promiscuous with Mn^2+^. Ksp11411I is more PT specific with Ni^2+^. EcoWI and Ksp11411I cleave fully- and hemi-PT modified oligos, while Bsp305I cleaves only fully modified ones. EcoWI forms a dimer in solution and cleaves more efficiently in the presence of two modified sites. In addition, we demonstrate that EcoWI PT-dependent activity has biological function: EcoWI expressing cells restrict dnd^+^ GpsAAC modified plasmid strongly, and GpsGCC DNA weakly. This work establishes a framework for biotechnology applications of PT-dependent restriction endonucleases (PTDRs).

## Introduction

DNA backbone phosphorothioation (PT or ps) is an alternative to DNA base methylation for distinguishing host from invading DNA in bacteria. Modified DNA has sulfur atoms in the *pro*-R (or Rp) conformation (Zhou et al., 2005). Sequence-dependent PT modifications are incorporated by the DndABCDE (DndA-E) enzyme complex. DndA is often replaced by a cysteine desulfurase such as IscS in *E. coli* (Liang et al., 2007; Wang et al., 2019). DndB serves as a negative regulator of the *dnd* operon and its inactivation can significantly increase the level of PT modification. PT modifications are usually introduced sequence specifically. So far, they have been found in four short sequence contexts: GpsGCC, GpsAAC/GpsTTC, GpsATC, or CpsCA/TGG (a hemi-modified PT site) (Wang et al., 2011, 2019). Only a fraction of target sites is modified, in either one or both strands. Less than 5% of available sites are PT modified in the dnd^+^ genomes of *E. coli* B7A (EcoB7A) and *Salmonella enterica* serovar Cerro 87 (SenC87) (Li et al., 2019). How the sites are selected for full, hemi- or no modification is not known.

In many cases, DNA phosphorothioation, analogously to methylation, acts as a mark of self, and provides an advantage for host over invading DNA. The traditional model suggests that both modifications alike should render DNA resistant to specialized endonucleases, in the case of phosphorothioation often diastereomer-selectively, as originally shown using synthetic substrates. The physiologically relevant nucleases are likely encoded by the *dndFGH* genes, which frequently accompany the *dndA-E* gene cluster. However, restriction activity of the DndFGH complex remains to be rigorously demonstrated *in vitro*. Moreover, it is not understood how phosphorothioation, that is usually very incompletely penetrant can protect from endonuclease cleavage. An alternative advantage for phosphorothioated over unmodified DNA, which avoids this paradox, has been proposed recently. In archaea, phosphorothioation becomes a requirement for DNA replication in the presence of PbeABCD genes. The DndCEDA – PbeABCD complex then serves as a phage exclusion (attenuation) system in the absence of apparent phage DNA degradation (Xiong et al., 2019).

In some cases, the role of PT modification can be reversed, presumably because invading phages or other mobile DNA have acquired the modification for protection, and because bacteria that do not use phosphorothioation of their own genomes have evolved to interpret this feature as a mark of an invader. Phosphorothioation-dependent restriction endonucleases (PTDRs) form a subgroup of the MDREs (Liu et al., 2010). ScoMcrA, the first enzyme shown to have PT-dependent endonuclease activity contains SRA, SBD and HNH domains (Liu et al., 2010). At least in theory, PTDRs may co-exist with dnd^+^ modification clusters if the PT modified sites have different sequence contexts.

SRA domains are found in prokaryotic and eukaryotic DNA binding proteins (Hashimoto et al., 2008; Han et al., 2015) and have a preference for DNA containing 5-methylcytosine (5mC), 5-hydroxymethylcytosine (5hmC), or glucosyl-5-hydroxymethylcytosine (g5hmC) (Horton et al., 2014a). On their own, the SRA domains are monomeric and have a single pocket for the modified DNA base (Hashimoto et al., 2008). In order to interact with this base, SRA domains have to extract it from the DNA stack, by nucleotide flipping (Hashimoto et al., 2008). Detailed pocket preferences vary among the domains. For example, the SRA domain from UHRF2 preferentially binds 5hmC. The SRA domain comprising PvuRts1I/AbaSI are most effective on DNA containing g5hmC, followed by 5hmC, but act poorly on DNA containing 5mC or C (Janosi et al., 1994; Szwagierczak et al., 2011; Horton et al., 2014a; Shao et al., 2014). *In vitro*, the isolated SRA domains additionally bind 5mC better than C (Kazrani et al., 2014). Some SRA domains sense cytosine modifications only in specific, frequently non-palindromic and partially degenerate sequence contexts (Horton et al., 2014b; Sasnauskas et al., 2015).

Sulfur binding domains (SBDs) are widespread in prokaryotic DNA binding proteins (Liu et al., 2018), but have so far not been found in eukaryotes. As the name indicates, they have a preference for sulfur in the Rp configuration in the DNA backbone. Like SRA domains, SBD domains are monomeric on their own and have a single, hydrophobic pocket for the sulfur atom, lined by apolar methyl or methylene groups of neutral or basic residues, and surrounded by positive charge. Both hydrophobicity and positive charge in the vicinity are likely to contribute to the preference for the modification. Sulfur is more hydrophobic than oxygen, and the negative charge is concentrated on the sulfur in O,O-dialkyl phosphorothioates, whereas it is delocalized between the non-bridging oxygen atoms for O,O-dialkyl phosphates (Frey and Sammons, 1985). The recent crystal structure of the SBD domain of ScoMcrA shows considerable protein-DNA base contacts in the vicinity of the sulfur atom (Liu et al., 2018), suggesting that at least some SBD domains bind PT in sequence context dependent manner.

HNH domains are endonuclease domains that are very widespread in prokaryotes, but rarely found in eukaryotes (Jablonska et al., 2017). The domains are named after moderately conserved residues that were present in the founding members. The nuclease core motif is composed of a β-hairpin followed by an α-helix, and one divalent metal ion (Me). This has given rise to the alternative and more informative description of this nuclease group as ββα-Me endonucleases. HNH (ββα-Me) enzymes have diverse requirements for the catalytic metal ion: some work with a wide variety of divalent metal cations, whereas others have more specialized requirements. For example, wild-type (WT) *Kpn*I is active in Mg^2+^, Mn^2+^, and Ca^2+^. High-fidelity *Kpn*I variants with decreased star activity have altered divalent cation requirements (Vasu et al., 2013). Another HNH enzyme, HpyAV prefers Ni^2+^ for catalytic activity (Chan et al., 2010). Many HNH endonucleases contain one or several zinc finger motifs, and therefore require Zn^2+^ ions for folding and structural integrity. HNH domains tend to dimerize, generating nucleases with two active sites that can make double-stranded (ds) cuts in DNA (Sokolowska et al., 2009). However, there are also monomeric HNH domains, such as colicin E9, which act as nickases on dsDNA (Pommer et al., 2001).

Here, we report an experimental characterization of selected SBD-SRA-HNH and SBD-HNH endonucleases. We verified that the SBD-SRA-HNH endonuclease ScoMcrA has a 5mC/5hmC dependence that is strongly relaxed in Mn^2+^ buffer. The phosphorothioate-dependent SBD-HNH endonuclease SprMcrA could be converted into a non-specific DNA nicking enzyme by N-terminal deletion in the SBD domain, demonstrating the role of the SBD domain in sulfur sensing. We further discovered five active PT-dependent and sequence-specific REases cleaving DNA at a distance from the modified sites. We tested EcoWI activity on synthetic substrates with one or two PT modified sites (hemi- or full-modification). We studied the cleavage directionality of hemi-modified oligoduplexes (GpsTTC) for EcoWI and Ksp11411I endonucleases in reference to the GpsT dinucleotide. We performed gel filtration chromatography to confirm that EcoWI forms dimers in solution. Finally, we confirmed the biological role of EcoWI, in restriction of incoming PT-modified DNA in plasmid transformation. The gene neighborhood analysis indicated that PT-dependent restriction systems are rarely coupled with dnd^+^ modification systems. This work provides a detailed study of DNA backbone modification-dependent restriction systems and a framework for future application of PTDRs in recombinant techniques.

## Materials and Methods

### Strains, Enzymes, and Expression Vector

DNA restriction and modification enzymes were provided by New England Biolabs, Inc (NEB). *E. coli* K NEB Turbo (Dcm^+^ RecA^+^) and *E. coli* B C2566 (T7 Express, Dcm^–^) strains were obtained from Dr. Elisabeth Raleigh’s collection (NEB). The IMPACT^*TM*^ protein expression and purification system (pTXB1 vector and chitin beads) was provided by NEB. The target gene (PCR product or synthetic gene blocks from IDT) was inserted into pTXB1 (*Nde*I and *Xho*I digested) in fusion with intein and a chitin binding domain (CBD) by using a HiFi DNA fragment assembly kit from NEB. Assembled plasmids plus insert were transferred into NEB Turbo or C2566 competent cells and transformants plated on rich agar plates with ampicillin (100 μg/ml) selection. Plasmid DNA transformation was carried out according to the manufacturer’s protocol (NEB). Inserts were sequenced to confirm the WT coding sequence. Target protein was purified through a chitin affinity column and DTT cleavage in the elution buffer (16–32 h).

### Protein Size-Exclusion Chromatography

Samples were injected 70 microliters per run onto a Superdex 200 10/300 GL (24 ml bed volume; GE Healthcare) and run at 4°C and 0.38 ml/min in standard column buffer (100 mM NaCl, 20 mM Tris–HCl, (pH 8), 1 mM DTT, 0.1 mM EDTA, 5% glycerol). Elution volume was averaged from multiple independent runs with purified EcoWI protein (2.7 mg/ml). The partition coefficient (*K*_av_) of EcoWI was determined by *K*_av_ = (*v*_e_ – *v*_o_)/(*v*_t_ – *v*_o_) where *v*_e_ is the elution volume, *v*_o_ is the void volume of the column and *v*_t_ is the total column volume. *v*_o_ and *v*_t_ were determined empirically by the addition of blue dextran and DTT, respectively, to the sample. A standard curve was created by obtaining the *K*_av_ value of the standard proteins carbonic anhydrase (29 kDa), ovalbumin (43 kDa), conalbumin (75 kDa), and Blue Dextran (GE Healthcare) run under the same condition, as described previously (Xu et al., 2011).

### Restriction Buffers and Modified/Unmodified DNA Substrates

Restriction buffers (NEBuffers) 1.1 (low salt), 2.1 (medium salt), 3.1 (high salt), and CutSmart^*TM*^ (buffer 4 + BSA)^1^ were used in all digestions unless specified otherwise. NEBuffer 1.1 (restriction buffer 1.1) contains 10 mM bis-Tris-propane-HCl, 10 mM MgCl_2_, 0.1 mg/ml BSA, pH 7.0 at 25°C. NEBuffer 2.1 (restriction buffer 2.1) contains 50 mM NaCl, 10 mM Tris–HCl, 10 mM MgCl_2_, 0.1 mg/ml BSA, pH 7.9 at 25°C. NEBuffer 3.1 (restriction buffer 3.1) contains 100 mM NaCl, 50 mM Tris–HCl, 10 mM MgCl_2_, 0.1 mg/ml BSA, pH 7.9 at 25°C. CutSmart buffer contains 50 mM KAc, 20 mM Tris-Ac, 10 mM MgAc_2_, 0.1 mg/ml BSA, pH 7.9 at 25°C. To test divalent cation requirement of a particular REase, the buffer containing 10 mM Tris–HCl, pH 7.5, 50 mM NaCl, and 1 mM DTT was used and supplemented with various divalent cation salts such as MnCl_2_ (1 mM) and CoCl_2_ (1 mM). 5hmC-modified PCR DNA fragments were amplified from pBR322 by Q5^®^ or Phusion^®^ DNA polymerase with 5hmdCTP replacing dCTP in dNTP mix (Zymo Research). Unmodified PCR fragments were amplified from pBR322 by 1x Master Mix with Q5^®^ or Phusion^®^ DNA polymerase and dNTP.

### Phosphorothioate (PT) Modified dnd^+^ Plasmids

The genomic DNA of *E. coli* B7A and *Salmonella enterica* Cerro 87 contains backbone PT modification in the GpsAAC/GpsTTC sequence context. In contrast, *Pseudomonas fluorescens* Pf0-1 gDNA contains palindromic GpsGCC modified sites (Wang et al., 2011). The *dnd* gene clusters responsible for PT modification of DNA backbone from *E. coli* B7A, *S. enterica* Cerro 87, *P. fluorescens* Pf0-1 (referred to as dnd^+^ (EcoB7A), dnd^+^ (SenC87), and dnd^+^ (Pfl), thereafter) were cloned into pUC19-derived expression vectors pLacZZ (constructed and provided by Z. Zhu, NEB) or pRRS (Skoglund et al., 1990). The *dnd* operons including PT-modification genes coding for B, C, D and E subunits (∼5 kb) were PCR amplified with Q5 DNA polymerase and cloned into pLacZZ (*Nde*I-*Bam*HI) or pRRS (*Pst*I-*Sma*I). Plasmids pLacZZ-dnd^+^ (EcoB7A, 10.4 kb), pLacZZ-dnd^+^ (SenC87, 10.4 kb), and pRRS-dnd^+^ (Pfl, 9.9 kb) were transferred into a methylase-deficient ER2796 *E. coli* strain. Plasmids pLacZZ-dnd^+^ (EcoB7A) and pLacZZ-dnd^+^ (SenC87) contained PT modification in the GpsAAC/GpsTTC sequence context, while pRRS-dnd^+^ (Pfl) contained GpsGCC modified sites.

The presence of PT modifications was verified by nuclease digestion and subsequent MS analysis. Total DNA (2 μg; a mixture of *E. coli* host genomic DNA and plasmid DNA) or 5 μg dnd^+^ plasmid DNA were treated by P1 nuclease and calf intestine alkaline phosphatase (Sigma-Aldrich). Nucleosides were purified by chromatography through filtration columns (10 kDa cut off).

To map the cut sites of REases, 12 synthetic primers were used to sequence uncut and cleaved plasmid dnd^+^ (SenC87), which are shown in Supplementary Table S1A. In control digestions, all three dnd^+^ plasmids were cleaved by *Hpa*II (CCGG) and MluCI (AATT) as expected, since the dnd^+^ modified sites do not overlap with the REases targets (data not shown). We used the dnd^+^ plasmid DNA for digestions and *in vivo* restriction assays (plasmid DNA transformation into restriction-proficient cells). It has been shown previously that over-expression of dnd clusters in *E. coli* modifies approximately 20% of the potential PT sites (Crooks et al., 2004). Similar modification levels were detected by PacBio sequencing and EcoWI digestion/sequencing (detailed PT site mapping results will be published elsewhere).

### Mapping of Cut/Nick Sites by DNA Sequencing

DNA run-off sequencing was carried out to map the cut/nick sites in restricted plasmids, using a BigDye^*TM*^ terminator V3.1 cycle DNA sequencing kit from ABI (Thermo Fisher Scientific).

Taq DNA polymerase adds an extra adenine base at the nucleotide (nt) position where the template is broken (due to cleavage introduced by REase or nicking enzyme), thus creating G/A, C/A, or T/A doublets. The A/A doublet is underrepresented in partial digestions, unless there is sharp drop-off after the A peak. In some cases, the A/A doublet was inferred from an extra high A peak when compared to the uncut template DNA. Cut/nick site consensus sequences were compiled by WebLogo server^2^. Sequencing primers for the dnd^+^ plasmid are listed in Supplementary Table S1A.

### PT-Modified Oligonucleotide Cleavage Assay

PT-modified PCR DNA was amplified from pBR322 using Q5 DNA polymerase, primers, and a dNTP pool containing 50% dATP/50% α-phosphorothioate-dATP (Trilink Biotechnologies), dCTP, dGTP, and dTTP or a dNTP mixture containing 50% dCTP/50% α-phosphorothioate-dCTP (Trilink Biotechnologies), plus dATP, dGTP, and dTTP. 100% of α-phosphorothioate-dATP replacing dATP or α-phosphorothioate-dCTP replacing dCTP in dNTP pool failed to produce any PCR products. Synthetic duplex oligonucleotides (60mer oligo, HPLC purified) containing PT modifications GpsAAC (complement GpsTTC or GTTC) with or without 5′ FAM label were purchased from IDT. The set included fully PT-modified oligos (GpsTTC/GpsAAC); unmodified oligos (GTTC/GAAC); hemi-PT-modified oligos (GpsTTC/GAAC) or (GTTC/GpsAAC) (Supplementary Table S1B). The hemi-modified oligos were used to test directionality of cleavage relative to the GpsT modified dinucleotide. The cleavage products were analyzed by capillary gel electrophoresis (CE assay) and DNA fragments (peaks) visualized by Peakscan software. To test cleavage efficiency on modified oligos with 0, 1, and 2 modified sites (e.g., one unmodified site plus second site with hemi- or full-PT modification; two sites with hemi- or full-PT modifications), another set of duplex oligos was synthesized (IDT) (Supplementary Table S1C). The digested products were treated with *E. coli* exonuclease I to remove single-stranded DNA (37°C for 20 min). Digestions were terminated by the addition of Proteinase K (37°C for 15 min). Cleavage products were resolved in 15% PAG-Urea gel (Thermo Fisher Scientific) and stained with SYBR gold and imaged on Typhoon imager (GE Healthcare).

## Results

### PT Modifications in dnd^+^ Plasmids

Nuclease-resistant GpsA, GpsT, and GpsG dinucleotides were detected by liquid chromatography-coupled tandem quadrupole mass spectrometry (triple Quad LC/MS-MS analysis), as described elsewhere (18). The PT detection parameters are shown in Supplementary Table S2 and the presence of PT-modified dinucleotides is summarized in Supplementary Table S3. The standard GpsA, GpsT, and GpsG dinucleotides were used as positive controls (Supplementary Figure S1). The exact numbers of fully- or partially modified GpsA, GpsT, and GpsG sites in the plasmids were unknown. The modified dinucleotides from six samples are shown in Supplementary Figure S2. The identical dinucleotides from three original bacterial genomes were also detected (data not shown).

### SBD-SRA-HNH Endonuclease ScoMcrA

**FIGURE 1:**
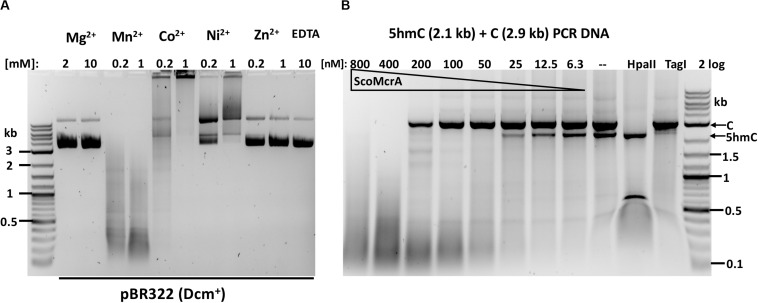
ScoMcrA divalent cation requirements and activity on mixed PCR fragments. **(A)** pBR322 [Dcm^+^, 1 μg (7 nM)] was digested by 50 nM of ScoMcrA in 50 mM NaCl buffer, supplemented with different divalent cations. Digestion in Co^2+^ ions formed a brown precipitate (some DNA was stuck in the loading well) even after Proteinase K treatment. The banding pattern in Mn^2+^ buffer suggests cleavage specificity. **(B)** ScoMcrA digestion of two PCR fragments (2.9 kb unmodified DNA and 2.1 kb 5hmC-containing DNA). *Hpa*II digests unmodified DNA only; TagI, preferentially cleaves 5hmC-modified DNA. 2 log: 0.1–10 kb DNA ladder (NEB).

ScoMcrA was named by analogy to EcoKMcrA, to highlight the shared endonuclease domains and the endonuclease activity against 5mC-modified DNA (Liu et al., 2010). However, the domain structures of EcoKMcrA and ScoMcrA are clearly different (Liu et al., 2018). EcoKMcrA consists of an N-terminal 5hmC binding domain, termed NEco, and an HNH catalytic domain. ScoMcrA (Sco4631) contains N-terminal “head”, SBD (phosphorothioate binding), SRA (5mC and 5hmC recognition) and C-terminal HNH endonuclease domains. ScoMcrA was purified by chitin and heparin chromatography and tested for activity using 5mC- or 5hmC-modified DNA, PT-modified plasmids and a PCR fragment as substrates. Consistent with the previous report (Liu et al., 2010), ScoMcrA is most active in the Mn^2+^ buffer (0.2 to 1 mM MnCl_2_), inactive in the Mg^2+^ buffer or in the presence of EDTA and partially active in the Co^2+^ buffer. The enzyme has strong nicking activity in the Ni^2+^ buffer (Figure 1A). The result of ScoMcrA digestion of mixed PCR DNA (C + 5hmC) is shown in Figure 1B. At high enzyme concentration (0.8 and 0.4 μM of protein vs 6.2 nM DNA), ScoMcrA cleaved both modified and unmodified DNAs almost indiscriminately (lanes 1-2). Upon further dilution (at 200-12.5 nM range), the enzyme preferentially cleaved the 5hmC-modified PCR fragment (lanes 3-7). In control experiments, *Hpa*II digested unmodified PCR fragment and TagI digested modified fragment, as expected. The above result suggests that ScoMcrA is specific for modified DNA only in a narrow range of protein concentrations and is prone to non-specific (star) activity in Mn^2+^ buffer. However, the activity on unmodified DNA is only relevant *in vitro* since ScoMcrA could be over-expressed in *E. coli* cells without significant toxicity, which implies tight regulation of its activity *in vivo* (fusion with intein and CBD may also attenuate the activity inside cells).

In agreement with the *in vitro* data, *E. coli* C2566 carrying *scoMcrA* gene restricted T4gt phage (5hmC) at 1.6 × 10^3^-fold compared to the empty vector under non-induced condition (i.e., no IPTG added). In contrast, the ScoMcrA expressing strain restricted λ_*vir*_ phage only ∼3-fold.

Our results, together with the previous data showing that the ScoMcrA expressing cells restricted 5mC modified DNA in plasmid DNA transformation (Liu et al., 2010), indicate that the enzyme is a *bona fide* MDRE specific toward 5mC and 5hmC modified DNA.

We next examined ScoMcrA activity on Dcm^+^ or Dcm^–^ pBR322 in Mn^2+^ buffer and mapped the cut sites in both substrates. ScoMcrA was able to cleave/nick both modified and unmodified pBR322 at the protein concentration range of 800 to 6 nM (and ∼7 nM DNA) (Supplementary Figures S3A,B). Clear banding patterns appeared in digestion with diluted enzyme (at 12 and 6 nM protein), suggesting that it had certain sequence preference. We mapped the cut/nick sites by direct sequencing of the digested DNA. The sequence logos (consensus sequence) of modified DNA (pBR322-Dcm^+^ or pBRFM^+^, 16 sites) and unmodified DNA (pBR322-Dcm^–^, pUC19-Dcm^–^, 42 sites) show sequence preference for ACN↓GT (also relaxed sequences AYN↓RT and RYN↓RY). The cleavage sites in the modified plasmids were mostly clustered near the methylated bases [C5mCWGG (Dcm) or G5mCNGC (M. *Fnu*4HI)] (Supplementary Figure S3C and data not shown), suggesting that the SRA domain binds to the modified cytosines and licenses cleavage nearby. The cleavage of unmodified plasmid is not clustered to the CCWGG or GCNGC sites (Supplementary Figure S3D).

We also tested ScoMcrA activity on plasmids carrying *dnd* modification gene clusters and PT-modified PCR fragment (by incorporation of α-PT-dATP in PCR). Due to the non-specific activity in Mn^2+^ buffer, the result was not conclusive (data not shown). This is consistent with a previous report that ScoMcrA shows strong non-specific endonuclease activity in 1 h digestion (Liu et al., 2010). Preferential digestion of modified DNA was detected only for 5 min reactions. Our results suggest that ScoMcrA is not suitable for mapping of PT-modified sites *in vitro* due to its substantial star activity in Mn^2+^ buffer at high enzyme concentration. It remains to be investigated whether binding proficient, but catalytically deficient ScoMcrA variants can be used to enrich PT-modified DNA *in vitro*.

### SBD-HNH Endonuclease SprMcrA

The combination of SBD and HNH domains is frequent and over 900 fusion homologs are found in a wide variety of phylogenetically distant bacteria in protein databases (data not shown).

**FIGURE 2:**
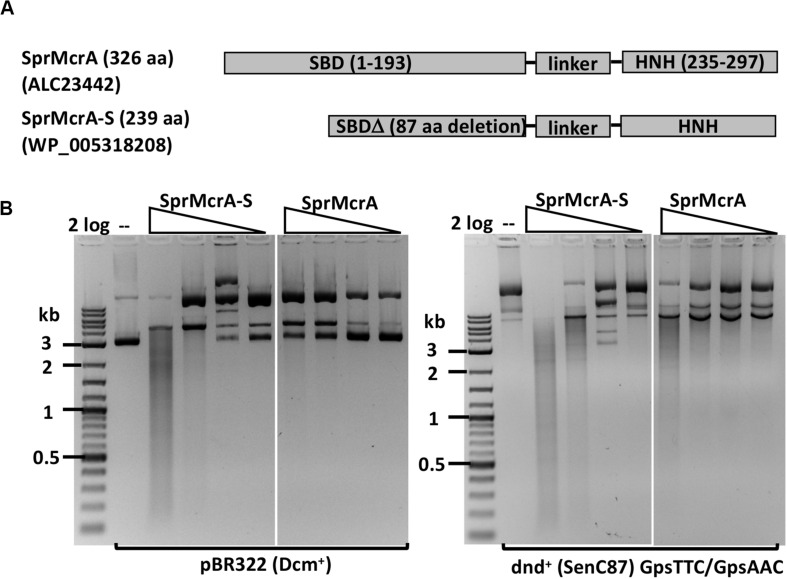
SprMcrA and SprMcrA-S (S for short truncated form) domain organization and activity assays. **(A)** Schematic diagram of SprMcrA and SprMcrA-S (SBD, linker region and HNH). **(B)** SprMcrA-S and SprMcrA digestion of pBR322 and dnd^+^ plasmids (SenC87) in Mn^2+^ buffer.

We selected SprMcrA, which features the SBD-HNH domain combination (GenBank ID ALC23442), and its shorter version with 87 aa deletion in the SBD domain (SprMcrA-S, GenBank ID WP_005318208), for further study. The domain organization of SprMcrA and SprMcrA-S is shown in Figure 2A. SprMcrA was shown to specifically cleave phosphorothioated DNA, at wobbled distance from the site of modification (Yu et al., 2018). SprMcrA and SprMcrA-S were overexpressed in *E. coli* as intein-CBD fusions and purified by chitin affinity chromatography. SprMcrA was further purified from a heparin column (Supplementary Figure S4A). The protein yield was lower for SprMcrA-S (∼50% of the full-length enzyme) probably due to its toxicity (non-specific nicking activity and loss of self-inhibition). The endonuclease activity of the two proteins was assayed in the presence of various divalent metal cations. Both were active in Mn^2+^ buffer and partially active in Co^2+^ buffer as previously reported (data not shown) (Yu et al., 2018). SprMcrA-S (Δ87 aa, N-terminal deletion variant) displayed partial endonuclease activity on pBR322 and dnd^+^ plasmids in Mn^2+^ buffer. The full-length enzyme showed slightly higher activity on dnd^+^ plasmid with GpsAAC/GpsTTC (Figure 2B). To examine sequence specificity of the truncated SprMcrA-S enzyme, we performed DNA sequencing of the partially digested pBR322. Supplementary Figure S5A shows three nicking sites (CG↓GT or GG↓GT, the down arrows indicate the nicked strand). The 5′ SS↓RT 3′ consensus sequence (Supplementary Figure 5B) was compiled from 47 nicking sites by WebLogo (Crooks et al., 2004). The nicking sites are reminiscent of the ones introduced by phage-encoded enzymes involved in DNA nicking and packaging (Xu and Gupta, 2013; Kala et al., 2014), suggesting that the HNH domains may be evolutionarily related. We conclude from the above results that the N-terminal 87 aa form part of the SprMcrA domain that recognizes PT-modified DNA. Similar observation was first demonstrated in a previous work (Yu et al., 2018). Removal of this N-terminal domain converts the enzyme to a non-specific nicking endonuclease (NEase). The original SprMcrA paper reported that some downstream sequences were nicked by the enzyme near the PT-modified sites (Yu et al., 2018), suggesting that SprMcrA may have both dsDNA cleavage and nicking activity. The sequence specificity of the HNH domain of SprMcrA may partially explain why some of the modified sites in pUC19, phosphorothioated by passing through dnd^+^ *E. coli* host, were not cleaved efficiently (Yu et al., 2018).

We next performed the *in vivo* restriction assay by dnd^+^ plasmid transformation into SprMcrA expressing cells. C2566 [pACYC184-SprMcrA] cells (uninduced) weakly restricted incoming dnd^+^ plasmid (GpsAAC/GpsTTC) 4 to 6-fold compared to cells carrying the empty vector (data not shown). This weak restriction activity may be due to poor expression and/or low endonuclease activity. It has been reported that WT SprMcrA carries the H-N-R catalytic residues in the HNH nuclease domain. Substitution of the Arg residue by Asn resulted in ∼40-fold increase of the enzyme activity (Yu et al., 2018).

### SBD-HNH Endonuclease EcoWI

**FIGURE 3:**
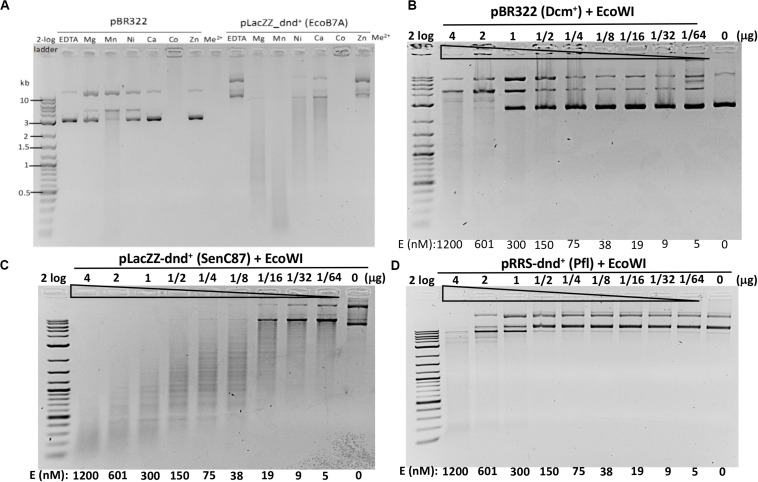
EcoWI endonuclease activity assays. **(A)** EcoWI (0.3 μM) digestion of pBR322 (Dcm^+^) and PT-modified pLacZZ-dnd^+^ (EcoB7A) in buffers supplemented with different divalent cations. **(B)** EcoWI digestion of pBR322 in Mg^2+^ buffer. **(C)** EcoWI digestion of dnd^+^ plasmid (SenC87) (GpsAAC/GpsTTC) in Mg^2+^ buffer. **(D)** EcoWI digestion of dnd^+^ plasmid (Pfl) (GpsGCC) in Mg^2+^ buffer.

Since ScoMcrA and SprMcrA endonucleases display either non-specific (ScoMcrA) or low endonuclease activity (SprMcrA) in Mn^2+^ buffer, we set out to screen SprMcrA homologs for more desirable properties. Conserved domain analysis by BlastP, Phyre2, and PROMALS3D indicated that EcoWI and a large number of ORFs in GenBank contain SBD domains and HNH nuclease domains, but lack the SRA domain (data not shown). We over-expressed and purified EcoWI and six other ORFs with moderate amino acid sequence similarity (Table 1). Supplementary Figure S4B shows the purified EcoWI endonuclease from chitin and heparin chromatography. EcoWI was active on dnd^+^ plasmid (EcoB7A) in buffers supplemented with Mg^2+^, Mn^2+^, Ni^2+^, or Ca^2+^. The activity in Co^2+^ buffer was inconclusive due to tight DNA binding and formation of a brown precipitate and the DNA getting aggregated and stuck in the loading well. EcoWI displayed non-specific activity on pBR322 (Dcm^+^) in Mn^2+^ buffer and low nicking activity in Mg^2+^ or Ni^2+^ buffers (Figure 3A). Thus, EcoWI and SprMcrA have different divalent cation requirements. SprMcrA is inactive in Mg^2+^ buffer. EcoWI in turn is similar to most Type IIM REases that require Mg^2+^ as a cofactor for catalytic activity.

**TABLE 1:** Summary of enzymatic properties of SBD-HNH endonucleases expressed, purified and characterized in this work.

Enzyme	Specific activity (U/mg)	Divalent cation dependence	Preferred dnd^+^ plasmid or PCR DNA substrate	Cut site (Type IIS and M)	Yield/L of induced cells	Amino acid similarity to EcoWI (%)	Sequence identity to EcoWI (%)
EcoWI	1000^#^	Mg^2+^	GpsAAC/GpsTTC	GpsAAC N7↓/N6↑	∼10 mg/L	100	100
Ksp11411I	500	Ni^2+^	GpsAAC/GpsTTC	GpsAAC N5↓/N4↑	∼8 mg/L	41.4	28.1
Bsp305I	500	Mg^2+^	GpsAAC/GpsTTC	GpsAAC N6↓/N(4-5)↑***	∼1 mg/L	43.7	28.1
Mae9806I	<500	Mn^2+^	GpsAAC/GpsTTC	GpsAAC N(8-10)↓/N(8-9)↑***	∼1 mg/L	45.7	27.3
Bsp48385I	<500	Mn^2+^	Low activity	Not determined	∼0.5 mg/L	46.9	28.4
SprMcrA *	<500	Mn^2+^	GpsAAC/GpsTTC	GpsAAC N(11-13)↓/N(10-12)↑	∼6 mg/L	27.4	18.5
SprMcrA-S	<500	Mn^2+^	No PT dependence	SS↓RT nicking sites	∼3 mg/L		
Sau43800I	<500	Mn^2+^	GpsAAC/GpsTTC	GpsAAC N(8-9)↓/N(7-8)↑	∼2 mg/L	47.0	31.3
Hba180I	<500	Mn^2+^	Active on α-S-dAMP PCR**	Not determined	∼1 mg/L	43.8	27.8

^#^Unit definition: 1 unit is the amount of enzyme required to digest 1 μg of dnd^+^ plasmid DNA (GpsAAC/GpsTTC) into fragments of less than 1 kb at 37°C for 1 h in buffer B2.1. For Ksp11411I digestion, the Mg^2+^ is replaced by Ni^2+^ (0.1 to 1 mM) in the restriction buffer. *SprMcrA has a variable cleavage distance GpsAAC N(11-13)/N(10-12) (Yu et al., 2018). **2’-Deoxyadenosine-5’-O-(1-thiotriphosphate) (alpha thiol dATP or 1-thio-dATP) 50% + dATP 50% in dNTP mix in PCR DNA amplification by Q5 DNA polymerase. ***The asymmetric cleavage range of the two DNA strands likely results from possible exonuclease activity.

We next examined the EcoWI PT modification-dependent activity in Mg^2+^ buffer. At high enzyme concentration the nuclease digested pBR322 lacking the PT modification (Figure 3B).

It cleaved pLacZZ-dnd^+^ (SenC87) substrate with modified GpsAAC/GpsTTC sites at 1.2 μM to 5 nM enzyme concentration (Figure 3C). EcoWI displayed low activity on pRRS-dnd^+^ (Pfl) plasmid containing GpsGCC/GpsGCC at high enzyme concentration (Figure 3D). It is not clear whether the cleavage took place near GGCC sites or other random sequences. EcoWI also cleaved PT-containing PCR DNA (with α-S-dATP incorporated into the PCR product) better than unmodified DNA (data not shown). Compared to computer generated digestion patterns generated by NEBcutter (Vincze et al., 2003), EcoWI achieved only partial cleavage of dnd^+^ plasmid probably due to the incomplete modification. Direct sequencing of EcoWI-digested dnd^+^ plasmid indicated that the enzyme cleaves next to its recognition sequence GpsAAC N7/N6 or GpsTTC N7/N6, generating one nt 3′ overhangs (Supplementary Figure S6). A significant number of nicked sites were also detected in both strands (the ratio of ds versus ss breaks was approximately 3:1 at high enzyme concentration). One to three DNA strand breaks were found near the PT modified sites, which might reflect the nature of partial modifications. In contrast to ScoMcrA and SprMcrA, EcoWI cleaved at a fixed distance from PT-modified sites. Therefore, the enzyme can be grouped into Type IIM (also Type IIS-like), whereas the other two nucleases, which cut PT-modified DNA with variable cleavage distances, were included in Type IV (Yu et al., 2018). We conclude that EcoWI is a Mg^2+^-dependent endonuclease that prefers GpsAAC/GpsTTC sequences. It displays lower activity on PT-modified DNA in a different sequence context (GpsGCC). The non-specific activity is strongly stimulated in Mn^2+^ buffer. EcoWI activity, if any, on GpsATC modified sites has not been tested. The cleavage specificity in Ni^2+^ and Ca^2+^ buffers also remains to be determined.

### EcoWI Restriction Activity *in vivo*

The biological function of the PT modification-dependent endonuclease activity is thought to be the restriction of incoming PT-modified (foreign) DNA. To test this hypothesis, we carried out a transformation experiment using resident pACYC184-*ecoWIR* and incoming dnd^+^ plasmids.

**FIGURE 4:**
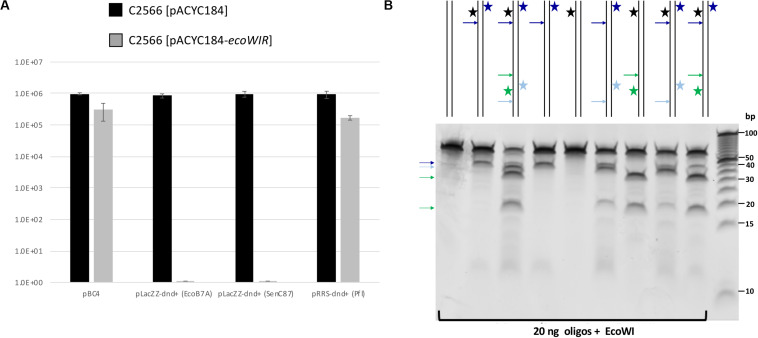
EcoWI *in vivo* restriction assay and digestion of modified duplex oligos. **(A)** Transformation efficiency of recipient cells C2566 [pACYC184] and [pACYC184-*ecoWIR*] with incoming dnd^+^ plasmids. No transformants were obtained for PT-modified dnd^+^ plasmids (EcoB7A or SenC87) into EcoWI-expressing strain (complete restriction). Control plasmid: pBC4 (pUC19 with adenovirus DNA insert). Control recipient cells: C2566 [pACYC184]. **(B)** EcoWI digestion of duplex oligos with one or two PT modified sites. 60mer oligoduplexes with PT modification in one or both strands indicated by a star (20 ng, ∼26 nM) were digested by EcoWI endonuclease (∼1.5 μM) in buffer 2.1 at 37°C for 30 min. Bold arrows indicate possible cleavage sites near the PT modified sites. 5 bp DNA size marker (10–100 bp, Thermo Fisher Scientific).

We first cloned the *ecoWIR* gene into pACYC184 under the control of the Tc promoter for constitutive expression. The expression of EcoWI was not lethal to the *E. coli* host (T7 Express C2566 strain), but the transformation efficiency dropped about 2- to 5-fold compared to the empty vector. We next tested the transformation of dnd^+^ plasmids into the EcoWI-expressing recipient cells. Three plasmids, pLacZZ-dnd^+^ (EcoB7A), pLacZZ-dnd^+^ (SenC87) and pRRS-dnd^+^ (Pfl) were transferred into C2566 [pACYC184] with comparable transformation efficiency (Figure 4A, solid bars). The transformation efficiency of pBC4 plasmid (pUC19 + adenovirus DNA insert) was comparable to that of the dnd^+^ plasmids. The endonuclease expression host C2566 [pACYC184-*ecoWIR*], however, could only be transformed with pBC4 or pRRS-dnd^+^ (Pfl) (gray bars). Zero transformants were obtained for EcoB7A or SenC87 dnd^+^ plasmids at 30–50 ng DNA per transformation and less than 20 transformants per μg of modified plasmid. The dnd^+^ (Pfl) plasmid may be partially restricted by the EcoWI endonuclease with approximately 5-fold drop in transformation efficiency. This result suggests that EcoWI may be able to bind to the PT-modified sites in dnd^+^ (Pfl) plasmid and slow down its replication.

### EcoWI Digestion of PT-Modified Oligonucleotides

To further confirm EcoWI preference for modified DNA, we used 5’-FAM labeled 60-bp oligoduplexes containing a single modified site (GpsAAC/GpsTTC) for digestion. The reaction products were resolved by capillary gel electrophoresis (CE assay). Supplementary Figure S7 shows that the modified oligos were only partially cleaved and that a relatively high enzyme concentration was required. EcoWI was capable of digesting both fully and hemi-PT modified oligos. Unlike dnd^+^ plasmids with *R*p-PT configuration, the synthetic oligos contained both *R*p and *S*p stereoisomers. It is known that PT-dependent endonucleases prefer to cleave modified DNA with *R*p configuration (Yu et al., 2018), which may explain partial reaction. EcoWI appeared to preferentially cleave downstream of hemi-modified GpsTTC/GAAC sequence (i.e., asymmetrically with respect to the GpsT modified dinucleotide). The mechanism of cleavage side preference (i.e., the position and orientation of the nuclease active site relative to the GpsT hemi-modified sequence) is unknown. It may be connected with the sequence preference of the HNH nuclease domain or simply an artifact of partial digestion.

### EcoWI Digestion of PT-Modified Oligos With One or Two Sites

Some Type IIS REases require two modified sites in DNA for efficient cleavage. To test the EcoWI dependence on multiple modified sites, we used a set of oligos containing one unmodified and one modified site (either hemi- or fully PT modified) and oligos with two hemi-modified sites. This is important for synthetic biology applications where it is essential to cut only the modified site and not the unmodified site. Figure 4B shows the EcoWI mediated cleavage of nine oligos with different configurations of 0–4 PT modifications in two sites. EcoWI displayed no activity on unmodified oligo. It had some low activity on oligo with one fully PT modified site close to the end of DNA. The activity was enhanced by the presence of the second PT modified site (four PT backbone modifications total). The substrates with two PT modified sites were cleaved more efficiently even when the two sites were hemi-modified. It is known that many Type IIS REases require two sites for efficient cleavage (Bath et al., 2002). The results of the experiments confirmed that EcoWI preferentially cleaves downstream of the PT-modification. The modification located close to the end of the oligo did not direct cleavage. Under the conditions used here, EcoWI cleavage was largely PT-dependent and not directed by unmodified sites (two GTTC sites are 20 bp apart).

### EcoWI Oligomerization State

ScoMcrA formed a dimer in the protein-DNA co-crystal structure (Liu et al., 2018) and SprMcrA in solution (Yu et al., 2018). We suspect that EcoWI may also exist as a dimer. The oligomerization state of EcoWI was investigated using gel-filtration chromatography on an analytical Superdex 200 column. The partition coefficient (*K*_av_) of EcoWI was compared to those of standard proteins to obtain the relative molecular weight (*M*_r_). The elution profile of EcoWI from three separate runs contains a single peak with an average *M*_r_ of ∼54.4 kDa (Supplementary Figure S8). The monomeric EcoWI protein appears as a 30 kDa protein in SDS-PAGE, slightly smaller than the predicted size of 33.4 kDa. The molecular mass of its dimer is expected to be 60 to 66.8 kDa. From the gel-filtration result we concluded that EcoWI forms a dimer in solution. The physical properties of EcoWI in the presence of specific and non-specific DNAs remain to be analyzed.

### Ksp11411I

**FIGURE 5:**
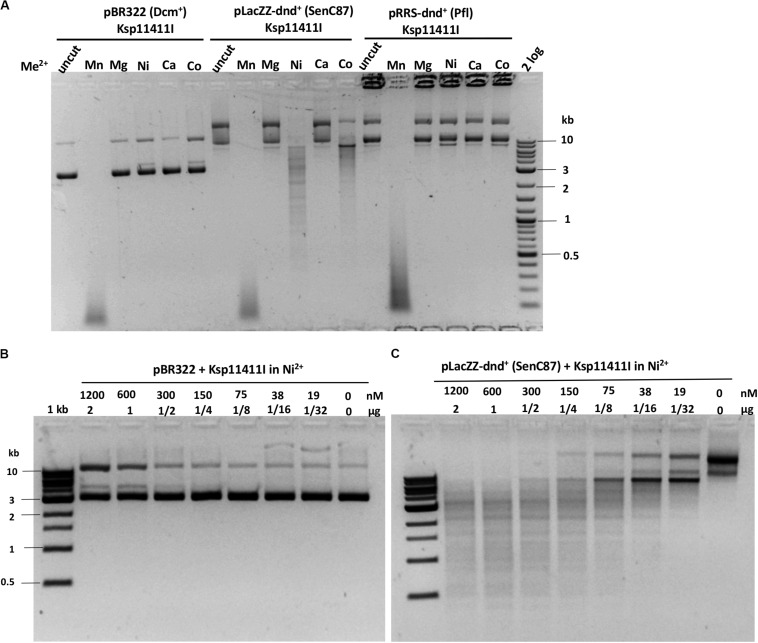
Ksp11411I divalent cation requirement and activity in Ni^2+^ buffer on methylated and phosphorothioated plasmids. **(A)** Ksp11411I mediated digestion of pBR322 (Dcm^+^), dnd^+^ (SenC87) and dnd^+^ (Pfl) plasmid in medium salt buffer supplemented with different divalent cations. **(B)** Ksp11411I digestion of pBR322 (Dcm^+^) in Ni^2+^ buffer (0.1 mM Ni^2+^). **(C)** Ksp11411I digestion of dnd^+^ plasmid (SenC87) in the same buffer as in **(B)**. The relative selectivity for dnd^+^ plasmid (SenC87) over pBR322 was estimated at 8 to 16-fold.

We evaluated six more EcoWI homologs with 41–47% aa sequence similarity (28–31% identity) which carry both SBD and HNH endonuclease domains (Table 1). Ksp11411I endonuclease was partially purified from the chitin column and the contaminating host gDNA was removed by incubating with DEAE beads in a high salt buffer. The partially purified Ksp11411I protein is shown in Supplementary Figure S4C. In Mn^2+^ buffer, Ksp11411I was active on dnd^–^ and dnd^+^ plasmid substrates (Figure 5A, Supplementary Figure S9). Ksp11411I showed a preference for PT-modified DNA upon dilution in Ni^2+^ buffer. In these conditions, the enzyme showed poor activity on pBR322 or dnd^+^ (Pfl) DNA (Figure 5AB), but strong activity on dnd^+^ plasmid (SenC87) (Figure 5C). Ksp11411I was partially active in Co^2+^ and inactive in Mg^2+^ buffer. Therefore, we decided to use Ni^2+^ buffer for cut site determination [so far only one other Type IIS REase HpyAV is known to prefer Ni^2+^ as a cofactor for activity (Chan et al., 2010)]. To map the cleavage sites, we sequenced the Ksp11411I digested dnd^+^ plasmid. One dsDNA cut site (GpsAAC N5↓/N4↑) and two nicking sites (GpsTTC N5↓ or GpsAAC N5↓) are shown in Supplementary Figure S10. Ksp11411I cleaved both fully and hemi PT-modified FAM-labeled oligoduplexes (Supplementary Figure S11). The enzyme preferentially cut hemi-modified oligos (GpsTTC) upstream of the PT site. We conclude that Ksp11411I requires Ni^2+^ (0.1 mM) as a cofactor for PT-dependent cleavage and displays strong non-specific activity in Mn^2+^ buffer. The distance between the PT-modified site and cleavage site is shorter (N5/N4) than that of EcoWI (N7/N6), but both enzymes generate one nucleotide 3′ overhangs. The directionality of cleaving hemi-modified sites (GpsTTC) is also different for the two enzymes.

### Bsp305I

**FIGURE 6:**
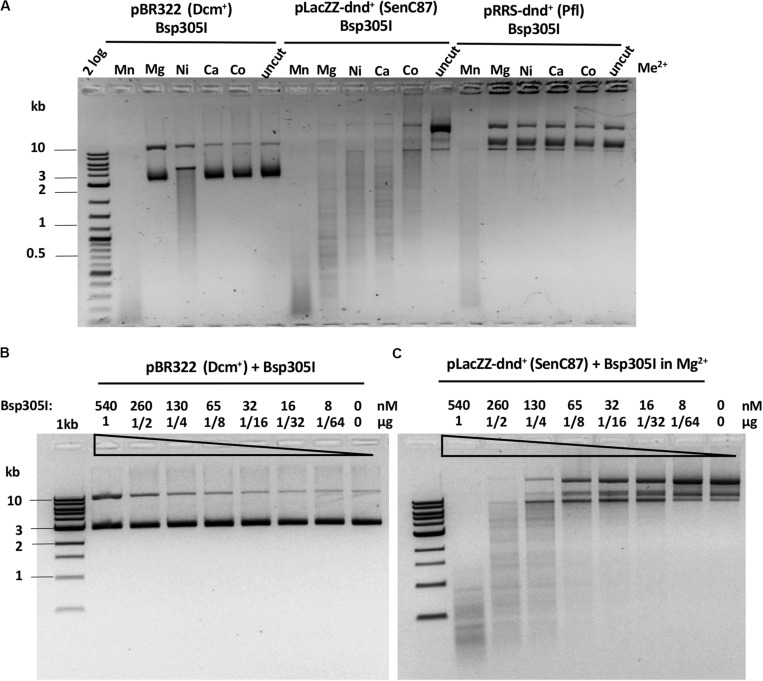
Bsp305I divalent cation requirement and restriction activity in Mg^2+^ buffer on methylated and phosphorothioated plasmids. **(A)** Bsp305I activity in a medium salt buffer supplemented with different divalent cations. **(B)** Bsp305I activity assay on pBR322 Dcm^+^ plasmid in Mg^2+^. **(C)** Bsp305I activity assay on dnd^+^ plasmid (SenC87) in Mg^2+^.

Bsp305I was purified by the same methods as Ksp11411I (Supplementary Figure S4C). Partially purified Bsp305I showed a broad divalent cation dependence and cleaved dnd^+^ plasmid (SenC87) in the presence of Mg^2+^, Mn^2+^, Ni^2+^, Ca^2+^, or Co^2+^ (Figure 6A). Similar to EcoWI and Ksp11411I, Bsp305I showed relaxed activity in Mn^2+^ buffer on pBR322 or dnd^+^ plasmid (Pfl) (Figure 6A). In the presence of Mg^2+^, however, the enzyme appeared to be more specific and cleaved dnd^+^ plasmid (SenC87) but showed poor activity on pBR322 (Figures 6B,C). DNA sequencing of the Bsp305I digested dnd^+^ plasmid indicated that it cleaves next to the modified sites: GpsTTC N6↓/N5↑, or GpsAAC N6↓/N(4–5)↑ (Supplementary Figure S12). The seemingly variable stagger might be the result of contaminating exonuclease digestion that nibbled away one base. However, we have not tested the exonuclease activity on unmodified DNA substrates. In CE assays on PT-modified duplex oligos, Bsp305I was able to cleave fully modified oligos in Mg^2+^ buffer. The enzyme was not very active on hemi-modified oligos (Supplementary Figure S13). We concluded that Bsp305I has moderate activity on dnd^+^ modified plasmid (SenC87) in buffer supplemented with Mg^2+^, Ni^2+^, or Ca^2+^. Hemi-PT-modified oligos are poor substrates for Bsp305I digestion.

### Bsp48385I, Mae9806I, Hba180I and Sau43800I

Bsp48385I, Mae9806I, and Hba180I endonucleases had very low activity on dnd^+^ plasmid. Bsp48385I discriminated between PT-modified and unmodified plasmid DNA only minimally in Mn^2+^ buffer (Supplementary Figure S14A). However, it preferred to cleave PT-modified over unmodified PCR substrates (Supplementary Figure S14B). Two major products were found in Bsp48385I digested unmodified DNA. Smaller cleavage products were derived from digestions of PT-modified PCR DNA. Sequencing of the Bsp48385I digested PCR DNA failed to generate specific cut site information (no clear doublets). Therefore, it is not clear whether the digestion of PCR DNA was due to PT-stimulated or modification-independent cleavage. The best PT modified DNA substrate, if any for Bsp48385I has yet to be determined.

Mae9806I displayed 2 to 4-fold preference for dnd^+^ DNA over pBR322 in Mn^2+^ buffer (Supplementary Figure S15). DNA sequencing of Mae9806I cleavage products (dnd^+^ plasmid) indicated that the cut/nick sites were variable at GpsAAC N(8–10)↓/N(8-9)↑ (Supplementary Figure S16). Again, we cannot rule out exonuclease contamination of the shorter stagger (N8) in the upper strand cut. The corresponding one base stagger would result in symmetric top and bottom strand cleavage at N9/N8 or N10/N9.

Sau43800I showed low, but detectable activity on dnd^+^ plasmid in Mn^2+^ buffer. It prefers to cleave dnd^+^ plasmid (SenC87) over pBR322 (Supplementary Figure S17A). DNA sequencing of the Sau43800I digested dnd^+^ plasmid indicated that the cut/nick sites are wobbled [GpsTTC N(8-9)/N(7-8)] with potential one nucleotide overhang N8/N7 or N9/N8 (Supplementary Figure S17B). Similarly, Hba180I displayed a low activity and cleaved PT-modified PCR DNA better than dnd^+^ plasmid or unmodified PCR DNA in Mn^2+^ buffer upon prolonged digestion (2 h), probably due to more frequent modified sites in the preferred substrate (Supplementary Figure S18).

### PROMALS3D: Multiple Sequence Alignment of EcoWI and Its Homologs

Secondary structure prediction and multi-sequence alignment of active EcoWI homologs indicated that the five PT-dependent REases can be divided into three regions (or domains) (Supplementary Figure S19). The N-terminal region contains the SBD, the putative PT and DNA sequence recognition domain (TRD) that harbors the conserved residues KPzzLL-zz–PF-L-W (*z* = I/L/V) that may contribute to the recognition of the sulfur atom and four bases (GTTC/GAAC) (Supplementary Figure S19). The C-terminal region contains the HNH catalytic domain with a potential zinc finger (CxxC and CxxH) that binds a zinc metal ion. The middle region is probably a variable linker that connects the SBD (TRD) and HNH nuclease domains and may serve to regulate the nuclease activity. This linker may be subjected to length variations to alter the cleavage distance. Supplementary Figure S20A shows part of the atomic structure of ScoMcrA SBD domain in complex with the PT-modified oligo. Multiple sequence alignment of the PT-dependent REases in the SBD domain (ClustalX) is shown in Supplementary Figure S20B, where the relatively conserved residues (HR or HK and YP) for sulfur recognition are highlighted in boxes.

### Genomic Neighborhood Analysis

If SBD-HNH endonucleases cleave PT-modified DNA, their coding genes should not co-occur with *dndABCDE* clusters of the same sequence specificity. In particular, unlike *dndFGH* genes, SBD-HNH endonuclease genes should not be enriched, and perhaps be underrepresented in the vicinity of *dndABCDE* genes. Orthologs of the nine studied SBD-HNH endonucleases were retrieved using the psi-BLAST web server. Either a single round of psi-BLAST with 500 allowed hits or three rounds of psi-BLAST with 1500 allowed hits were performed. After each round only the hits with query coverage above 90% were selected. The genomic neighborhoods of the hits were retrieved from the NCBI database with the NCBI E-utilities (with ∼70% efficiency)^3^, and/NBK179288/. Entrez Programming Utilities (E-utilities) Help). As the sets selected for the nine enzymes were overlapping, in total almost 3000 unique sequences were retrieved from the database. A first generous selection resulted in the preliminary set of ∼100 neighborhoods containing *dnd* or *dnd*-related genes. The resulting set was trimmed to the genes where the putative PT-dependent enzyme was less than four genes apart from a *dnd* (-related) gene. The set was then manually analyzed for the likelihood of the correct sequence assignment of the *dnd* gene, the character of the separating genes and the presence of another nuclease in the vicinity of the *dnd* gene cluster, which resulted in the selection of ∼65 unresolved cases. The genomic neighborhood analysis showed that less than 3.6% of all predicted PTDRs may be associated with a *dnd* modification cluster (Supplementary Table S4). The coexistences may be explained by either the *dnd* gene cluster being mutated or the PTDR being inactive, having low activity, or lacking PT-dependence. Alternatively, the targets of the modification-dependent endonuclease and the *dnd* complex may differ in DNA sequence context to avoid hazard to the host genome.

## Discussion

ScoMcrA is an SBD-SRA-HNH enzyme with an SRA domain that potentially directs specificity toward DNA containing modified cytosine bases, an SBD domain that may convey PT-dependence and an effector HNH nuclease domain. In this work we independently confirmed that the enzyme has 5hmC-stimulated activity on modified PCR DNA *in vitro* and modified T4gt phage DNA *in vivo*. The PT-stimulated activity of the enzyme was inconclusive in Mn^2+^ buffer. The ScoMcrA HNH domain may have certain sequence preference, most likely for RYN↓RY targets next to the modified sites. Some of the Dcm modified sites were poorly cleaved, probably reflecting the lack of RYNRY sites adjacent to modified cytosines (Liu et al., 2010).

SprMcrA is an SBD-HNH protein. We showed that 87 residues at the N-terminus of its SBD domain were required for PT-stimulated activity. Truncation of the SBD domain resulted in a non-specific nicking enzyme independent of PT modification. We evaluated seven further SBD-HNH endonucleases found in GenBank that were predicted to be PT-dependent by domain analysis. Five homologs were active on dnd^+^ plasmid and two homologs had low activity on PT-containing PCR DNA. EcoWI and Bsp305I were active in Mg^2+^ buffer and both had broad divalent cation requirement for catalysis. Ksp11411I was more specific for PT-modified DNA in Ni^2+^ buffer (this is the second example of REase that requires Ni^2+^ as a cofactor for specific activity). Mae9806I, Sau43800I, Bsp48385I, and Hba180I were active in Mn^2+^ buffer only (i.e., inactive in Mg^2+^), similar to SprMcrA and ScoMcrA. It is possible that the metal ion binding site (nuclease catalytic center) of EcoWI and Bsp305I is slightly different from that of the Mn^2+^-dependent REases (SprMcrA and ScoMcrA). Structure analysis of EcoWI will be desirable to compare the atomic structure of the catalytic sites. But the difference may be very subtle. Our previous work on *Kpn*I specificity showed that a single aa change altered metal ion binding: a conservative aa substitution of the metal-coordinating residue D148 to Glu results in the elimination of the Ca^2+^-mediated cleavage but imparting high cleavage fidelity with Mg^2+^. High cleavage fidelity of the variant D148E is achieved through better discrimination of the target site at the binding and cleavage steps (Vasu et al., 2013).

The active homologs shared the recognition sequence (GpsAAC/GpsTTC) and cleaved it leaving 1 nt 3′ overhangs with small variation in distance between the modification and the cut site [EcoWI: N7/N6, Ksp11411I: N5/N4, Bsp305I: N6/N5, Mae9806I: N(9-10)/N(8-9)], Sau43800I: N(8-9)/N(7-8), the ambiguous asymmetric cleavage sites were omitted).

We propose to include the SprMcrA and EcoWI-like enzymes in the Type IIM family since they require DNA backbone PT modification and cleave/nick near the modified sites. The oligoduplex digestions indicated that some enzymes (EcoWI and Ksp11411I) cut substrates with a single modified site. EcoWI activity may be enhanced by the presence of two or more sites, a property found among various Type IIS enzymes (Bath et al., 2002). Bsp305I cleaves fully PT modified oligos efficiently, and hemi-PT-modified oligos poorly. EcoWI and Ksp11411I cleave both fully and hemi-PT modified sites, but the directionality of the hemi-PT modified site dependent cleavage differs between the two enzymes. EcoWI prefers to cleave downstream and Ksp11411I upstream of the GpsT dinucleotide. The domain organization of EcoWI and its homologs is similar to the Type IIS *Mnl*I (CCTC N7/N6), that also carries the HNH catalytic domain (Kriukiene, 2006), suggesting that PT-stimulated REases may have evolved from Type IIS enzymes by the addition of PT modified DNA dependence.

### Biological Function of the dnd Modification Enzymes and PT-Dependent REases

The PT modifications of short DNA sequences (usually 3–4 bp) are thought to impair or completely block bacteria-encoded Type I, II, and III REases. PT backbone modifications can serve as a marker of “self”, and unmodified DNA of “foreign”. In this case, DndFGH endonuclease could form a complex with DndA-E to attack unmodified DNA, a restriction mechanism similar to the Type I R-M systems. However, partial modification of bacterial genomes by DndA-E might cause self-restriction. We still do not know how bacteria are able to solve this self-inflicting problem. Co-existence of the 6mA and C5 MTases that modify the GATC to G6mATC or GAT5mC with dnd^+^ gene clusters that modify the same sequence to GpsATC, may provide partial protection. Similarly, cytosine MTases (e.g., M. *Hae*III) that methylate the GGCC sequence can also cross-protect partially PT modified genomes (assuming the GpsGCC Dnd modification specificity). PT modifications in promoter and operator regions may also play a role in the regulation of gene expression, but not much supporting *in vivo* evidence has been reported. Alternative function of the sulfur atoms as receptors for cations, polyamines, and S-reactive radicals that would alter the DNA backbone structure remains to be investigated. PT modifications in bacteria under anaerobic conditions such as in human and animal guts remain to be investigated.

PT-dependent REases were thought to restrict PT-modified mobile genetic elements in horizontal gene transfers or to prevent DNA invasion. In this scenario, the PT modified sequences would serve as markers of “foreign”. Unmodified DNA or PT backbone modification in a different sequence context would serve as “self”. For example, *Streptomyces coelicolor* A3 contains only 4mC modified bases and no PT modifications in its genomic DNA (dnd^–^) (Lutz et al., 2019). ScoMcrA endonuclease encoded by its genome restricts dnd^+^ plasmid (GpsGCC/GpsGCC) coming from *Streptomyces lividans* that contains *dndA-E* modification genes (He et al., 2007). The restriction was not mediated by the 5mC/5hmC specific SRA domain since the plasmid DNA was isolated from Dcm-deficient cells. In this work we demonstrated that EcoWI endonuclease expressed in *E. coli* cells strongly restricts incoming plasmids dnd^+^ modified in a particular sequence context (GpsAAC/GpsTTC) and mildly restricts plasmid dnd^+^ modified in GpsGCC context. GpsATC modified substrates have not been tested. There is some evidence that SprMcrA is able to bind to a few PT-modified oligos in GpsR sequence context (*R* = A or G) (Yu et al., 2018). Therefore, the restriction (attenuation) by tight binding to PT-modified DNA (to slow down replication) is another possible mechanism to “restrict” foreign DNA. The recently discovered SspE nicking restriction system (nickase/NTPase), coupled with the SspABCD dnd modification enzymes, attenuated unmodified phages. SspE-mediated restriction was alleviated if the phages had been grown in dnd^+^ strain and PT-modified (CpsCA) (Xiong et al., 2020). Thus, the SspE nicking restriction system differs in specificity and type of DNA cleavage from the PTDR systems described in this work although both recognize PT backbone modifications.

Additional putative REases with SBD and HNH domains were found in GenBank and protein databases. More than 350 homologs of EcoWI (30% to 100% sequence identity) are present in *Enterobacteria* (*E. coli*, *Salmonella, Klebsiella, Enterobacter, Citrobacter*, and *Serratia*). Less homologous proteins are found in other bacteria. The number of putative PTDRs are increasing monthly from sequenced bacterial genomes and metagenomes. Screening these enzymes for PT-modification dependent activities may uncover new sequence specificities and cleavage distances and provide more tools for genetic engineers.

Recent structure analysis of SprMcrA SBD domain in complex with three PT-modified substrates (GpsAAC-congate site, GpsGCC and GpsATC-non-cognate sites) revealed the detailed interactions of the enzyme with the target DNA sequences and backbone modification through hydrogen bonding and electrostatic interactions. This information will provide clues in engineering SBD variants with new specificities and improved selectivity on PT-modified sites (Yu et al., 2020). It has been successfully demonstrated that star activity of REases (*Bam*HI, *Eco*RI, and *Kpn*I) can be significantly reduced by protein engineering (SYX, Z. Zhu, S.H. Chan, unpublished results) (Vasu et al., 2013) (US patent number 8673610).

### Gene Neighborhood Analysis of PTDR

Among the nine REases reported in this work. Only Bsp48385I is closely associated with *dnd* modification (*dndA-E*) and restriction (*dndFGH*) clusters and it is probably located on a mobile genetic element (a transposase is located in its proximity) (Supplementary Figure S21). Bsp48385I displays a low residual activity on plasmid DNA regardless of PT modification (Supplementary Figure S15). Therefore, we suspect that Bsp48385I will likely not cause self-restriction of PT modified host DNA when its expression is under tight regulation in its native system. The analysis of genomic neighborhoods of generously selected homologs of the studied enzymes indicates that the *dnd* gene clusters are present in their immediate vicinity in less than 3.6% of cases (Supplementary Table S4). The remaining outliers might result from non-functional *dnd* clusters, inactivating mutations in the HNH domains or misalignment of SBD and non-SBD domains. Alternatively, the SBD-HNH enzymes and the host Dnd modification proteins might have different sequence specificity and thus the problem of self-restriction should be diminished.

### Potential Application in Mapping of PT-Modified Sites in Bacterial Genome and Human Microbiome

EcoWI-like PT-dependent REases cleave DNA near PT modified GpsAAC/GpsTTC sites. EcoWI digested PT-modified plasmid or gDNA can be ligated to adaptors to construct libraries for Illumina sequencing. Short end sequence reads can be mapped to the reference genome to infer the PT-modified sites. The modified sites can be compared to those of PacBio sequencing and iodine-mediated cleavage mapping. This work is ongoing in our labs and the results will be published elsewhere. The non-specific star activity on unmodified sites warrants further study (e.g., reducing star activity/increasing relative selectivity on PT modified DNA by protein engineering) before these enzymes can be widely used in molecular biology applications. The dnd^+^ expression plasmid can be used as a positive selection vector for insert screening in conjunction with EcoWI-mediated restriction. For example, positive insert into the dnd^+^ genes (inactivation of the dnd^+^ modification cluster) would survive the EcoWI restriction and form colonies in transformation. Future study will be focused on screening new PTDRs that efficiently cleave GpsGCC or GpsATC modified sites. For example, by screening metagenome libraries that fail to form transformants in dnd^+^ (GpsGCC or GpsATC) expressing *E. coli* host. It is anticipated that EcoWI and related enzymes will provide additional tools in studies of PT-modification in metagenomes and microbiomes.

## Data Availability Statement

The raw data supporting the conclusions of this article will be made available by the authors, without undue reservation.

## Author Contributions

TL, HC, AF, VP, DH, BC, PD, MB, and SX generated and analyzed the experimental data. SX, HC, and MB wrote the manuscript with the assistance from TL, AF, DH, and PD. SX, PD, and MB conceived the idea. All authors contributed to the article and approved the submitted version.

## Conflict of Interest

TL, SX, AF, VP, and DH were employed by New England Biolabs, Inc., and declare that this study received funding from the New England Biolabs, Inc. The funder had the following involvement with the study: reporting results to the company management team for evaluation of possible patent application. No patent application was filed on this subject matter. The funder supports the decision to publish. The remaining authors declare that the research was conducted in the absence of any commercial or financial relationships that could be construed as a potential conflict of interest.

## Footnotes

1 www.neb.com

2 https://weblogo.berkeley.edu/

3 https://www.ncbi.nlm.nih.gov/books/NBK25499

## References

[B1] Bath A. J. Milsom S. E. Gormley N. A. Halford S. E. (2002). Many type IIs restriction endonucleases interact with two recognition sites before cleaving DNA. *J. Biol. Chem.* 277 4024–4033. 10.1074/jbc.m108441200 11729187

[B2] Chan S. H. Opitz L. Higgins L. O’Loane D. Xu S. Y. (2010). Cofactor requirement of HpyAV restriction endonuclease. *PLoS One* 5:e9071. 10.1371/journal.pone.0009071 20140205 PMC2816704

[B3] Crooks G. E. Hon G. Chandonia J. M. Brenner S. E. (2004). WebLogo: a sequence logo generator. *Genome Res.* 14 1188–1190. 10.1101/gr.849004 15173120 PMC419797

[B4] Frey P. A. Sammons R. D. (1985). Bond order and charge localization in nucleoside phosphorothioates. *Science* 228 541–545. 10.1126/science.2984773 2984773

[B5] Han T. Yamada-Mabuchi M. Zhao G. Li L. Liu G. Ou H. Y. (2015). Recognition and cleavage of 5-methylcytosine DNA by bacterial SRA-HNH proteins. *Nucleic Acids Res.* 43 1147–1159. 10.1093/nar/gku1376 25564526 PMC4333417

[B6] Hashimoto H. Horton J. R. Zhang X. Bostick M. Jacobsen S. E. Cheng X. (2008). The SRA domain of UHRF1 flips 5-methylcytosine out of the DNA helix. *Nature* 455 826–829. 10.1038/nature07280 18772888 PMC2602803

[B7] He X. Ou H. Y. Yu Q. Zhou X. Wu J. Liang J. (2007). Analysis of a genomic island housing genes for DNA S-modification system in Streptomyces lividans 66 and its counterparts in other distantly related bacteria. *Mol. Microbiol.* 65 1034–1048. 10.1111/j.1365-2958.2007.05846.x 17640271

[B8] Horton J. R. Borgaro J. G. Griggs R. M. Quimby A. Guan S. Zhang X. (2014a). Structure of 5-hydroxymethylcytosine-specific restriction enzyme. AbaSI, in complex with DNA. *Nucleic Acids Res.* 42 7947–7959. 10.1093/nar/gku497 24895434 PMC4081097

[B9] Horton J. R. Nugent R. L. Li A. Mabuchi M. Y. Fomenkov A. Cohen-Karni D. (2014b). Structure and mutagenesis of the DNA modification-dependent restriction endonuclease AspBHI. *Sci. Rep.* 4:4246. 10.1038/srep04246 PMC3946040 24604015

[B10] Jablonska J. Matelska D. Steczkiewicz K. Ginalski K. (2017). Systematic classification of the His-Me finger superfamily. *Nucleic Acids Res.* 45 11479–11494. 10.1093/nar/gkx924 29040665 PMC5714182

[B11] Janosi L. Yonemitsu H. Hong H. Kaji A. (1994). Molecular cloning and expression of a novel hydroxymethylcytosine-specific restriction enzyme (PvuRts1I) modulated by glucosylation of DNA. *J. Mol. Biol.* 242 45–61. 10.1006/jmbi.1994.1556 8078071

[B12] Kala S. Cumby N. Sadowski P. D. Hyder B. Z. Kanelis V. Davidson A. R. (2014). HNH proteins are a widespread component of phage DNA packaging machines. *Proc. Natl. Acad. Sci. U.S.A.* 111 6022–6027. 10.1073/pnas.1320952111 24711378 PMC4000778

[B13] Kazrani A. A. Kowalska M. Czapinska H. Bochtler M. (2014). Crystal structure of the 5hmC specific endonuclease PvuRts1I. *Nucleic Acids Res.* 42 5929–5936. 10.1093/nar/gku186 24634440 PMC4027163

[B14] Kriukiene E. (2006). Domain organization and metal ion requirement of the Type IIS restriction endonuclease MnlI. *FEBS Lett.* 580 6115–6122. 10.1016/j.febslet.2006.09.075 17055493

[B15] Li J. Chen Y. Zheng T. Kong L. Zhu S. Sun Y. (2019). Quantitative mapping of DNA phosphorothioatome reveals phosphorothioate heterogeneity of low modification frequency. *PLoS Genet.* 15:e1008026. 10.1371/journal.pgen.1008026 30933976 PMC6459556

[B16] Liang J. Wang Z. He X. Li J. Zhou X. Deng Z. (2007). DNA modification by sulfur: analysis of the sequence recognition specificity surrounding the modification sites. *Nucleic Acids Res.* 35 2944–2954. 10.1093/nar/gkm176 17439960 PMC1888814

[B17] Liu G. Fu W. Zhang Z. He Y. Yu H. Wang Y. (2018). Structural basis for the recognition of sulfur in phosphorothioated DNA. *Nat. Commun.* 9:4689. 10.1038/s41467-018-07093-1 PMC6224610 30409991

[B18] Liu G. Ou H. Y. Wang T. Li L. Tan H. Zhou X. (2010). Cleavage of phosphorothioated DNA and methylated DNA by the type IV restriction endonuclease ScoMcrA. *PLoS Genet.* 6:e1001253. 10.1371/journal.pgen.1001253 21203499 PMC3009677

[B19] Lutz T. Flodman K. Copelas A. Czapinska H. Mabuchi M. Fomenkov A. (2019). A protein architecture guided screen for modification dependent restriction endonucleases. *Nucleic Acids Res.* 47 9761–9776. 10.1093/nar/gkz755 31504772 PMC6765204

[B20] Pommer A. J. Cal S. Keeble A. H. Walker D. Evans S. J. Kuhlmann U. C. (2001). Mechanism and cleavage specificity of the H-N-H endonuclease colicin E9. *J. Mol. Biol.* 314 735–749. 10.1006/jmbi.2001.5189 11733993

[B21] Sasnauskas G. Zagorskaite E. Kauneckaite K. Tamulaitiene G. Siksnys V. (2015). Structure-guided sequence specificity engineering of the modification-dependent restriction endonuclease LpnPI. *Nucleic Acids Res.* 43 6144–6155. 10.1093/nar/gkv548 26001968 PMC4499157

[B22] Shao C. Wang C. Zang J. (2014). Structural basis for the substrate selectivity of PvuRts1I, a 5-hydroxymethylcytosine DNA restriction endonuclease. *Acta Crystallogr. D. Biol. Crystallogr.* 70 2477–2486. 10.1107/s139900471401606x 25195760 PMC4157451

[B23] Skoglund C. M. Smith H. O. Chandrasegaran S. (1990). Construction of an efficient overproducer clone of HinfI restriction endonuclease using the polymerase chain reaction. *Gene* 88 1–5. 10.1016/0378-1119(90)90052-s 2187744

[B24] Sokolowska M. Czapinska H. Bochtler M. (2009). Crystal structure of the beta beta alpha-Me type II restriction endonuclease Hpy99I with target DNA. *Nucleic Acids Res.* 37 3799–3810. 10.1093/nar/gkp228 19380375 PMC2699513

[B25] Szwagierczak A. Brachmann A. Schmidt C. S. Bultmann S. Leonhardt H. Spada F. (2011). Characterization of PvuRts1I endonuclease as a tool to investigate genomic 5-hydroxymethylcytosine. *Nucleic Acids Res.* 39 5149–5156. 10.1093/nar/gkr118 21378122 PMC3130283

[B26] Vasu K. Nagamalleswari E. Zahran M. Imhof P. Xu S. Y. Zhu Z. (2013). Increasing cleavage specificity and activity of restriction endonuclease KpnI. *Nucleic Acids Res.* 41 9812–9824. 10.1093/nar/gkt734 23963701 PMC3834813

[B27] Vincze T. Posfai J. Roberts R. J. (2003). NEBcutter: a program to cleave DNA with restriction enzymes. *Nucleic Acids Res.* 31 3688–3691. 10.1093/nar/gkg526 12824395 PMC168933

[B28] Wang L. Chen S. Vergin K. L. Giovannoni S. J. Chan S. W. DeMott M. S. (2011). DNA phosphorothioation is widespread and quantized in bacterial genomes. *Proc. Natl. Acad. Sci. U.S.A.* 108 2963–2968. 10.1073/pnas.1017261108 21285367 PMC3041111

[B29] Wang L. Jiang S. Deng Z. Dedon P. C. Chen S. (2019). DNA phosphorothioate modification-a new multi-functional epigenetic system in bacteria. *FEMS Microbiol. Rev.* 43 109–122. 10.1093/femsre/fuy036 30289455 PMC6435447

[B30] Xiong L. Liu S. Chen S. Xiao Y. Zhu B. Gao Y. (2019). A new type of DNA phosphorothioation-based antiviral system in archaea. *Nat. Commun.* 10:1688. 10.1038/s41467-019-09390-9 PMC6459918 30975999

[B31] Xiong X. Wu G. Wei Y. Liu L. Zhang Y. Su R. (2020). SspABCD-SspE is a phosphorothioation-sensing bacterial defence system with broad anti-phage activities. *Nat. Microbiol.* 5 917–928. 10.1038/s41564-020-0700-6 32251370

[B32] Xu S. Y. Corvaglia A. R. Chan S. H. Zheng Y. Linder P. (2011). A type IV modification-dependent restriction enzyme SauUSI from *Staphylococcus aureus* subsp. aureus USA300. *Nucleic Acids Res.* 39 5597–5610. 10.1093/nar/gkr098 21421560 PMC3141236

[B33] Xu S. Y. Gupta Y. K. (2013). Natural zinc ribbon HNH endonucleases and engineered zinc finger nicking endonuclease. *Nucleic Acids Res.* 41 378–390. 10.1093/nar/gks1043 23125367 PMC3592412

[B34] Yu H. Li J. Liu G. Zhao G. Wang Y. Hu W. (2020). DNA backbone interactions impact the sequence specificity of DNA sulfur-binding domains: revelations from structural analyses. *Nucleic Acids Res.* 10.1093/nar/gkaa574 [Epub ahead of print]. 32621606 PMC7470945

[B35] Yu H. Liu G. Zhao G. Hu W. Wu G. Deng Z. (2018). Identification of a conserved DNA sulfur recognition domain by characterizing the phosphorothioate-specific endonuclease SprMcrA from Streptomyces pristinaespiralis. *Mol. Microbiol.* 110 484–497. 10.1111/mmi.14118 30184284

[B36] Zhou X. He X. Liang J. Li A. Xu T. Kieser T. (2005). A novel DNA modification by sulphur. *Mol. Microbiol.* 57 1428–1438. 10.1111/j.1365-2958.2005.04764.x 16102010

