# Supplementary material for: Protein Domain Guided Screen for Sequence Specific and Phosphorothioate-Dependent Restriction Endonucleases
Source: Front Microbiol. 2020 Aug 18;11:1960. doi: 10.3389/fmicb.2020.01960 (PMC7461809; doi:10.3389/fmicb.2020.01960)
Supplement: Supplementary file 1 [file Data_Sheet_1.PDF]

Supplementary Materials: Suppl. Tables S1-S4 and Suppl. Figures S1-S21

Protein domain guided screen for sequence specific and phosphorothioate-dependent restriction endonucleases

Thomas Lutz[1], Honorata Czapinska[2#], Alexey Fomenkov[1#], Vladimir Potapov[1], Dan Heiter[1], Bo Cao[3,4], Peter Dedon[3], Matthias Bochtler[2,5] and Shuang-yong Xu*[1]

1. New England Biolabs, Inc. 240 County Road, Ipswich, MA 01938, USA.
2. International Institute of Molecular and Cell Biology, Trojdena 4, 02-109 Warsaw, Poland.
3. Department of Biological Engineering, Massachusetts Institute of Technology, Cambridge, MA 02139.
4. College of Life Science, Qufu Normal University, Qufu, Shandong 273165, China.
5. Institute of Biochemistry and Biophysics PAS, Pawinskiego 5a, 02-106 Warsaw, Poland.

# These authors contributed equally and may be considered as joint second author.

*Corresponding author
E-mail: xus@neb.com
Telephone: 1-978-380-7287
Fax: 1-978-921-1350

Running title: phosphorothioate-dependent restriction endonucleases

Suppl. Table S1. Synthetic single-stranded oligos (purchased from IDT).
A. DNA primers (5′ to 3′) used to sequence dnd⁺ (SenC87) plasmid near GAAC/GTTC sites.

F90	ATGTGCGCGGAACCCCTATTTGTTTATTTTTC
R600	TGGCCGCAGTGTTATCACTCATGGTTATGGC
F800	TCCCGGCAACAATTAATAGACTGGATGGAGG
R1400	TAACTACGGCTACACTAGAAGGACAGTATTTG
F3000	ATTGGCAGCTAGCGGCTAAAAAAGAGGTTT
R3600	ACAGGTGTTTCCACCAGCGTATCGGATGACA
F3600	GGTAGTAGATTTAATTAAGAAAACGATGTTGC
R4200	GCTTTATCTTTGGTGACTACGGTACAGGTC
F4600	GGTGGTTGACGATCAGTAGCGCTTTGAT
R5200	TTGTGCACCAGTGCCCCGAGTTGTTCAACAT

B. The PT-modified and unmodified oligos (5’-3’) with one site (GAAC/GTTC).

**1.** TCCACCGGGCTGCAGGAATTCGACTAG**GpsTTC**ATCGGGCGATACCGTCGACCTCGAGGATG
**2.** CATCCTCGAGGTCGACGGTATCGCCCGAT**GpsAAC**CTAGTCGAATTCCTGCAGCCCGGTGGA
**3.** TCCACCGGGCTGCAGGAATTCGACTAG*GTTC*ATCGGGCGATACCGTCGACCTCGAGGATG
**4.** CATCCTCGAGGTCGACGGTATCGCCCGAT*GAAC*CTAGTCGAATTCCTGCAGCCCGGTGGA

Fully PT-modified oligos: 1+2; unmodified oligos 3+4; hemi-PT-modified oligos 1+4 (GpsTTC/GAAC) or 2+3 (GTTC/GpsAAC). The hemi-modified oligos were used to test directionality of cleavage in relative to the GpsT modified dinucleotide. The cleavage products were analyzed by capillary gel electrophoresis (CE assay) and DNA fragments (peaks) visualized by Peakscan software (ABI/Thermo-Fisher).

C. Synthetic oligos with 0, 1, and 2 PT-modified sites (e.g., one unmodified site plus second site with hemi- or full-PT modification; two sites with hemi- or full-PT modifications).

5′ CTCGGC*GAAC*TCACTCTGGATGCTGTAGGC*GAAC*GCTTGGTTATGCCGGTACTGCCAGAC 3′<br>(two unmodified sequences *GAAC*, one site near the end N6 *GAAC*, the second site near the middle).
5′ GTCTGGCAGTACCGGCATAACCAAGC*GTTC*GCCTACAGCATCCAGAGTGA*GTTC*GCCGAG 3′<br>(two unmodified sequences *GTTC*, one site near the end *GTTC* N6, the second site near the middle).
5′ CTCGGC**GpsAAC**TCACTCTGGATGCTGTAGGC*GAAC*GCTTGGTTATGCCGGTACTGCCAGAC 3′<br>(one GpsAAC modified, second *GAAC* unmodified).
5′ GTCTGGCAGTACCGGCATAACCAAGC*GTTC*GCCTACAGCATCCAGAGTGA**GpsTTC**GCCGAG 3′<br>(one *GTTC* unmodified, second GpsTTC modified, GpsTTC N6)
5′ CTCGGC*GAAC*TCACTCTGGATGCTGTAGGC**GpsAAC**GCTTGGTTATGCCGGTACTGCCAGAC 3′<br>(one *GAAC* unmodified, second GpsAAC modified).
5′ GTCTGGCAGTACCGGCATAACCAAGC**GpsTTC**GCCTACAGCATCCAGAGTGA*GTTC*GCCGAG 3′<br>(one GpsTTC modified, second *GTTC* unmodified).
5′ CTCGGC**GpsAAC**TCACTCTGGATGCTGTAGGC**GpsAAC**GCTTGGTTATGCCGGTACTGCCAGAC 3′<br>(both sequences GpsAAC modified).
5′ GTCTGGCAGTACCGGCATAACCAAGC**GpsTTC**GCCTACAGCATCCAGAGTGA**GpsTTC**GCCGAG 3′<br>(both sequences GpsTTC PT modified, GpsTTC N6 is too short to be cleaved at the 3’ end).

Suppl. Table S2. Detection parameters in triple Quad LC/MS analysis of (nuclease resistant) PT-modified dinucleotides (1).

PT dinucleotides	Precursor ion	Product ion	Dwell	Fragmentor	Collision energy
d(GpsA)	597	136	200	120	17
d(GpsT)	588	152	200	110	17
d(GpsG)	613	152	200	123	29

Suppl. Table S3. Summary of PT-modified dinucleotides in total DNA (genomic and plasmid DNA), bacterial genomic DNA, and dnd^{+} plasmid DNA.

Sample name	GpsA	GpsT	GpsG
**Total DNA**			
ER2796 [pLacZZ dnd^{+} EcoB7A]	yes	yes	--
ER2796 [pLacZZ dnd^{+} SenC87]	yes	yes	--
ER2796 [pRRS dnd^{+} Pfl]	--	--	yes
**Bacterial genomic DNA**			
E. coli B7A (EcoB7A)	yes	yes	--
Salmonella enterica Cerro87 (SenC87)	yes	yes	--
Pseudomonas fluorescens Pf0-1 (Pfl)	--	--	yes
**Dnd^{+} plasmid DNA**			
pLacZZ dnd^{+} EcoB7A	yes	yes	--
pLacZZ dnd^{+} SenC87	yes	yes	--
pRRS dnd^{+} Pfl	--	--	yes

Suppl. Table S4. Gene neighborhood analysis of PT-dependent REases (PTDRs). A. Analysis of sequences obtained after three rounds of psi-BLAST and with manual outlier verification. "Outliers" correspond to predicted PT-dependent REases (PTDRs) in the vicinity of the putative dnd+ gene cluster. The maximum number of potential PTDRs located close to dnd+ gene clusters appeared to be 3.6% (102/2830). The cases where the PTDR and dnd+ genes were separated by more than four other genes and/or where the neighborhood could be explained by gene defects/presence of other nucleases in the vicinity were marked as resolved.

Enzyme<br>(GenBank accession number)	Number of ORFs tested	Outliers	un-resolved	resolved
ScoMcrA (WP_011029780.1)	24	0		
SprMcrA (REBASE)	688	3		3
SprMcrA (ALC23442)	35	0		
Ksp11411I (WP_030459191.1)		5		5
Bsp48385I (WP_088102990.1)	1078	14		14
Sau43800I (WP_121012085.1)	1021	11		11
EcoWI	730	8		8
Hba180I (MBC76123.1)		9		9
Bsp305I (WP_109358847.1)	1178	92	64	28
Mae9806I (WP_004160581.1)	1116	89	63	26
**TOTAL**	**2830**	**102**	**64**	**38**

B. Analysis of sequences obtained after one round of psi-BLAST and without manual outlier verification. The cases where the PTDR and dnd^+ genes were separated by more than four other genes were marked as resolved.

Enzyme<br>(GenBank accession number)	Number of ORFs tested	Outliers	un-resolved	resolved
ScoMcrA (WP_011029780.1)	17	0		
SprMcrA (ALC23442)	29	0		
Ksp11411I (WP_030459191.1)	303	3	2	1
Bsp48385I (WP_088102990.1)	378	9	3	6
Sau43800I (WP_121012085.1)	342	2	0	2
EcoWI	186	2	0	2
Hba180I (MBC76123.1)	320	22	21	1
Bsp305I (WP_109358847.1)	239	52	40	12
Mae9806I (WP_004160581.1)	279	72	55	17

Suppl. Fig. S1. Standard PT-modified dinucleotides GpsA and GpsT and GpsG in triple Quad LC/MS analysis (1).

GpsA

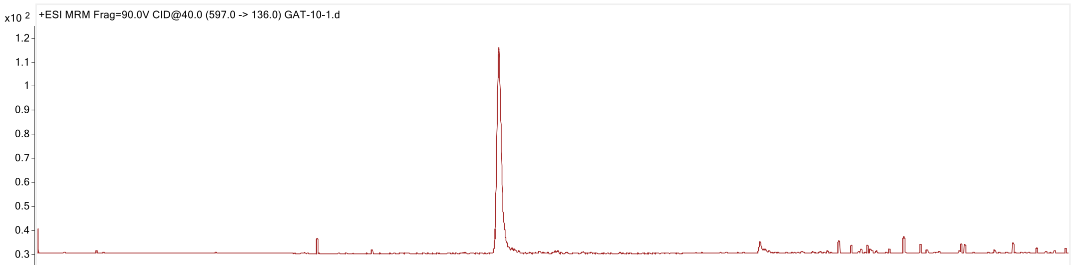

GpsT

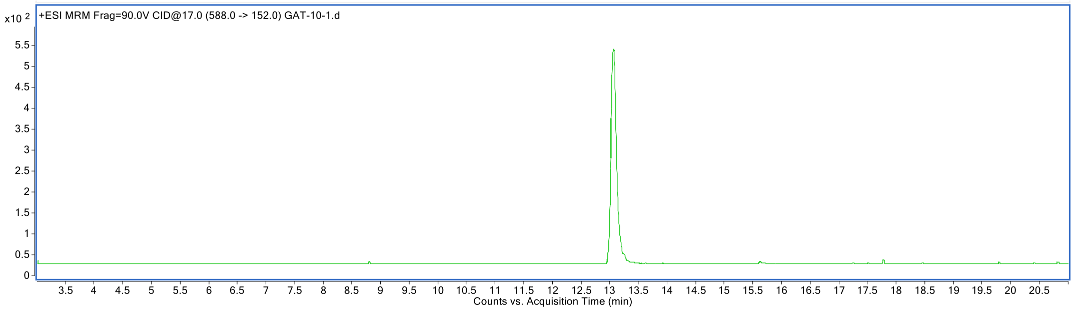

GpsG

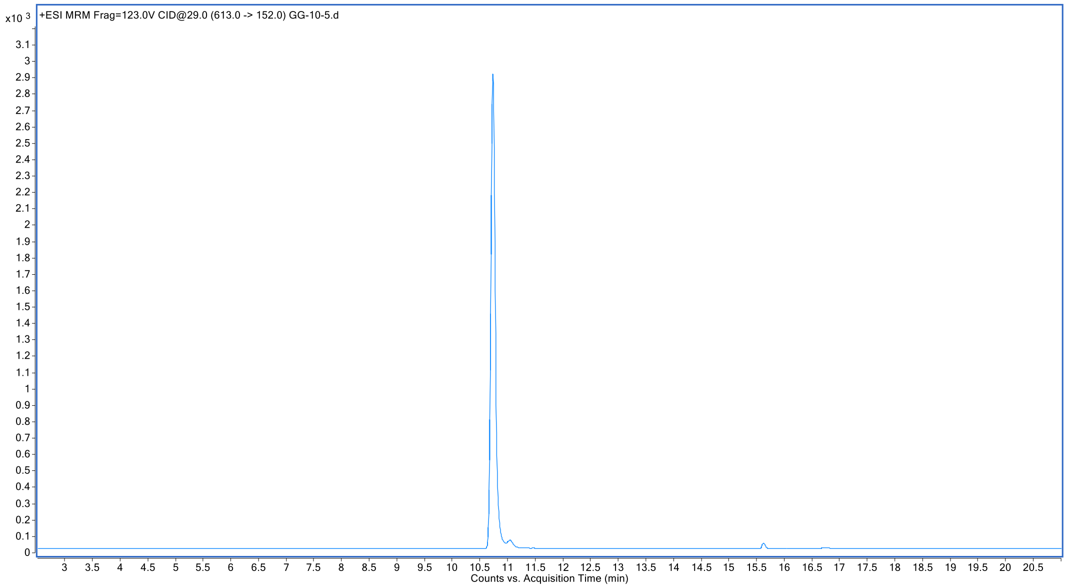

Suppl. Fig. S2. Triple Quad LC/MS analysis of total DNA (genomic and dnd$^+$ plasmid DNA), and dnd$^+$ plasmid DNA.

Total DNA: genomic DNA and dnd$^+$ EcoB7A plasmid (GpsA and GpsT)

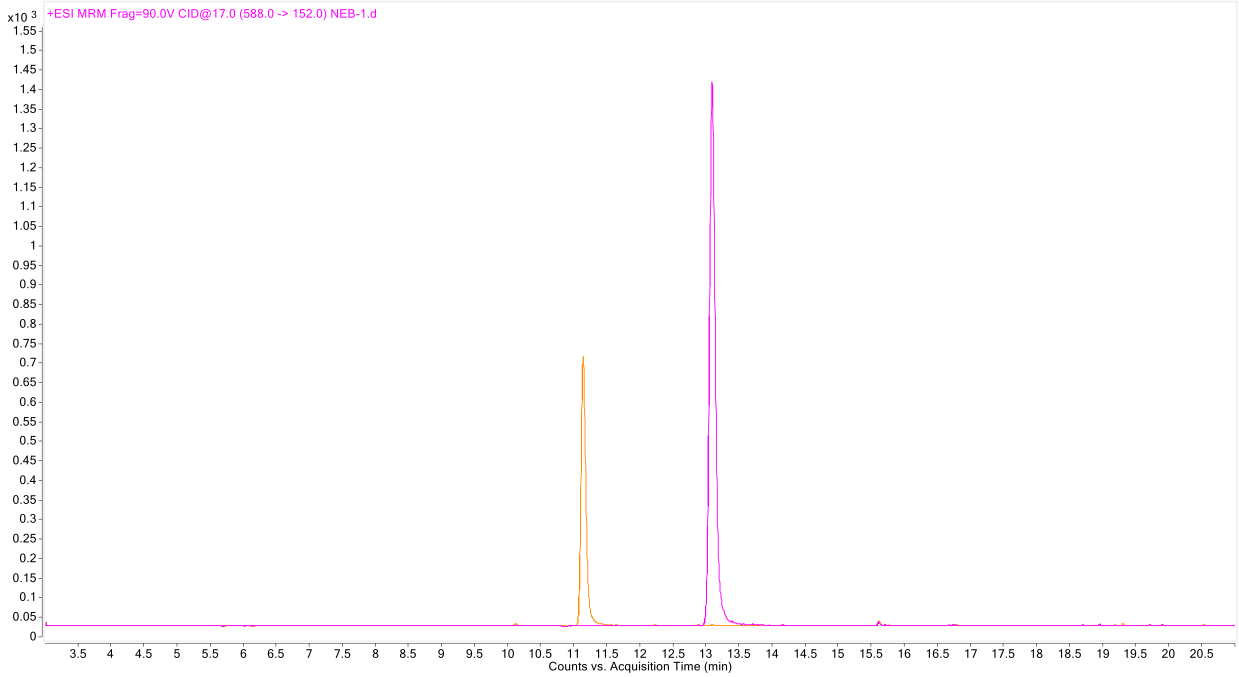

Dnd$^+$ EcoB7A plasmid (GpsA and GpsT)

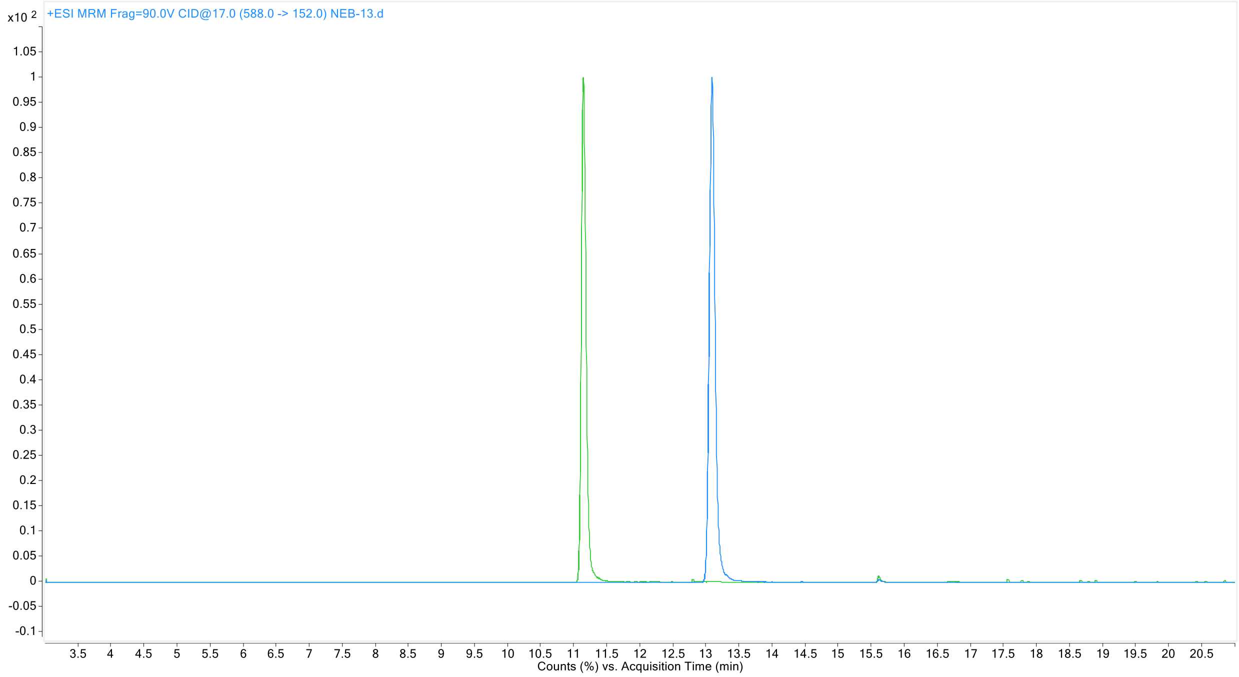

Total DNA: genomic DNA and dnd$^+$ SenC87 plasmid (GpsA and GpsT)

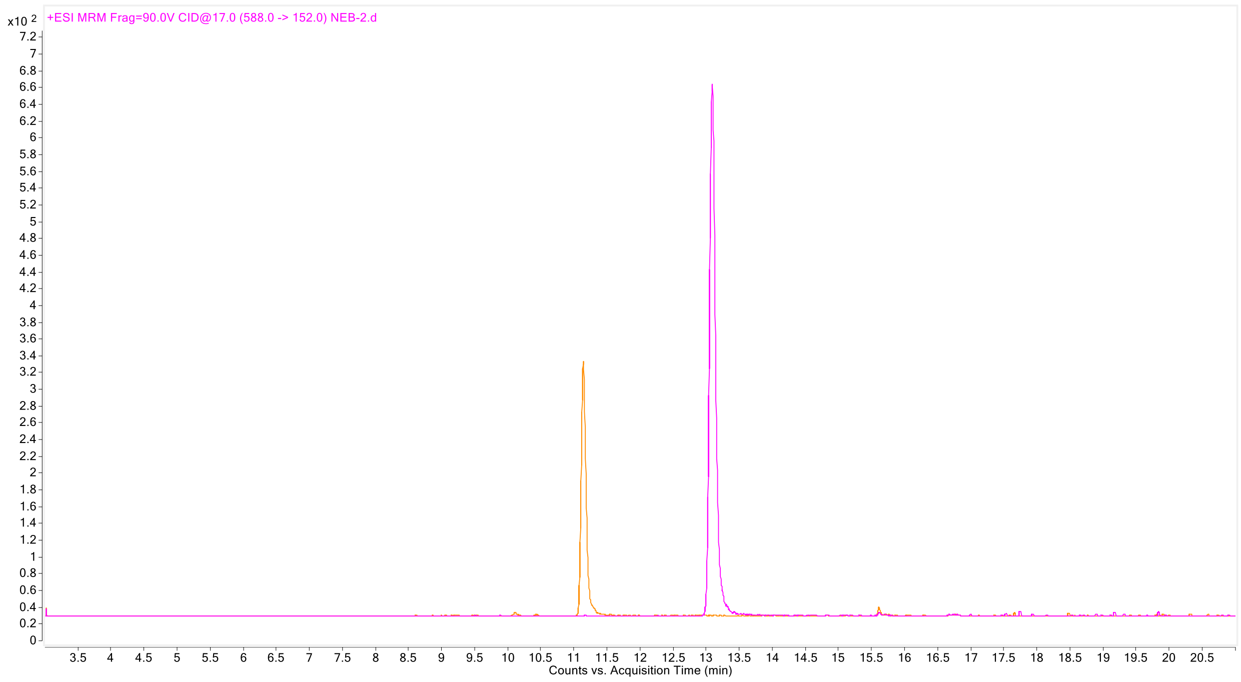

Dnd$^+$ SenC87 plasmid (GpsA and GpsT)

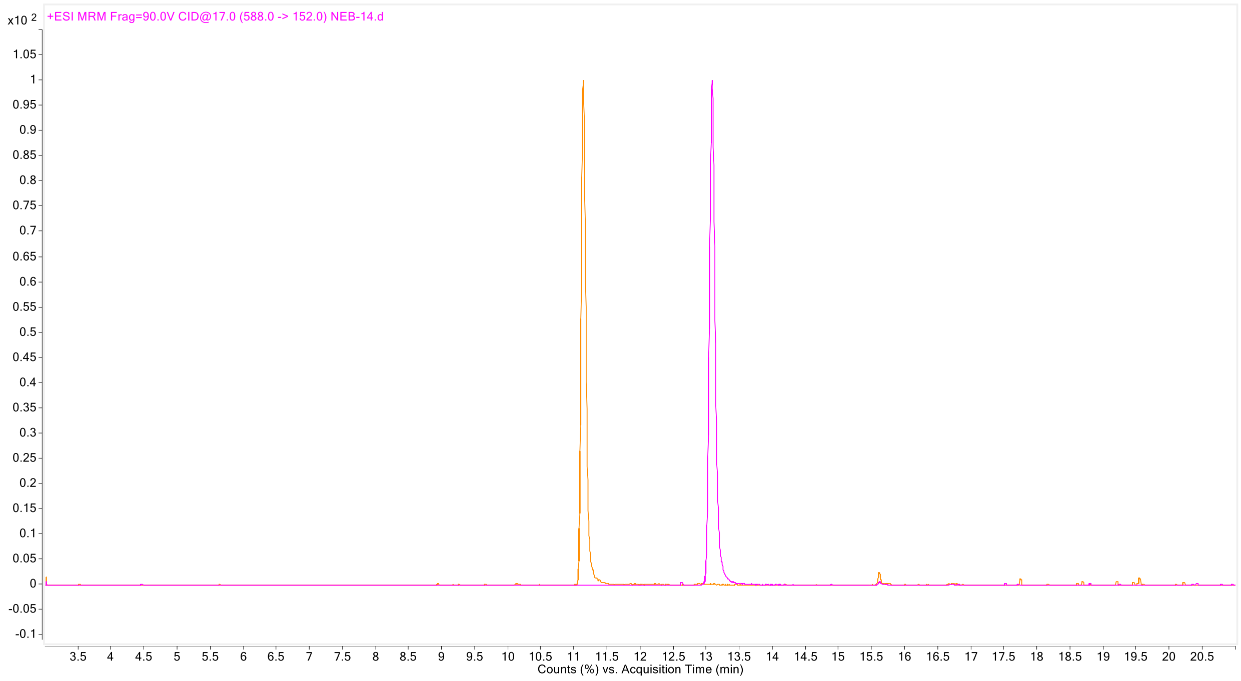

Total DNA: genomic DNA and dnd$^+$ Pfl plasmid (GpsG)

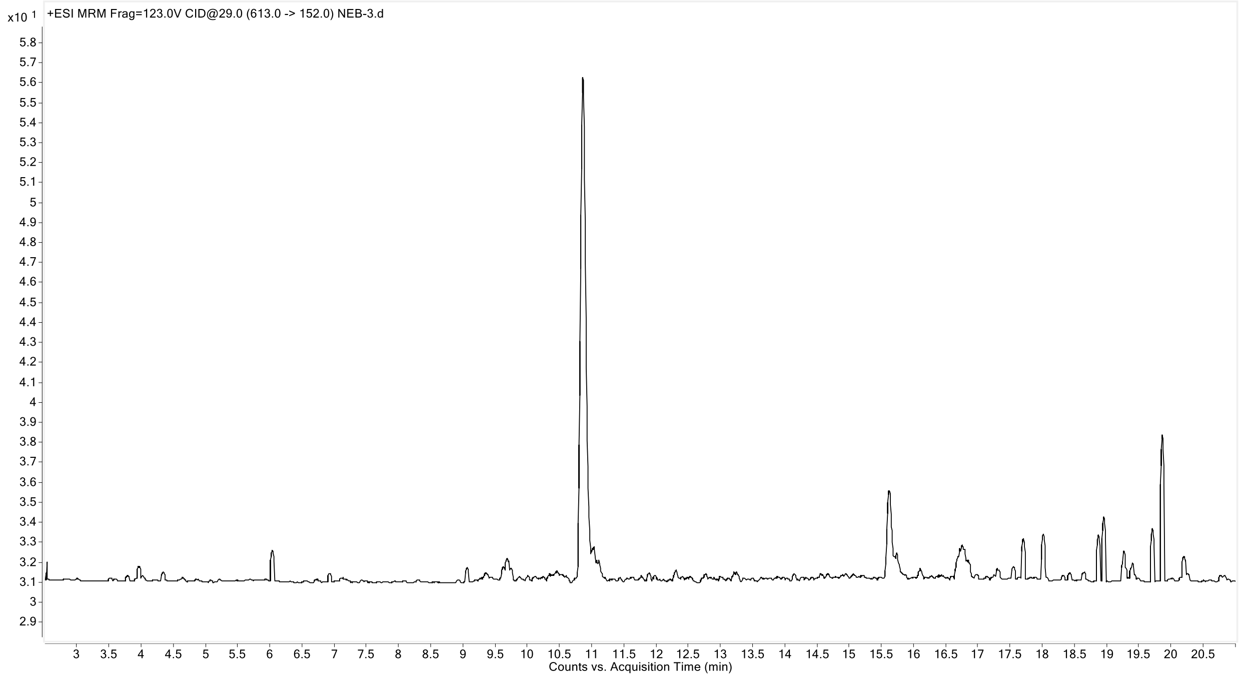

Dnd$^+$ Pfl plasmid (GpsG)

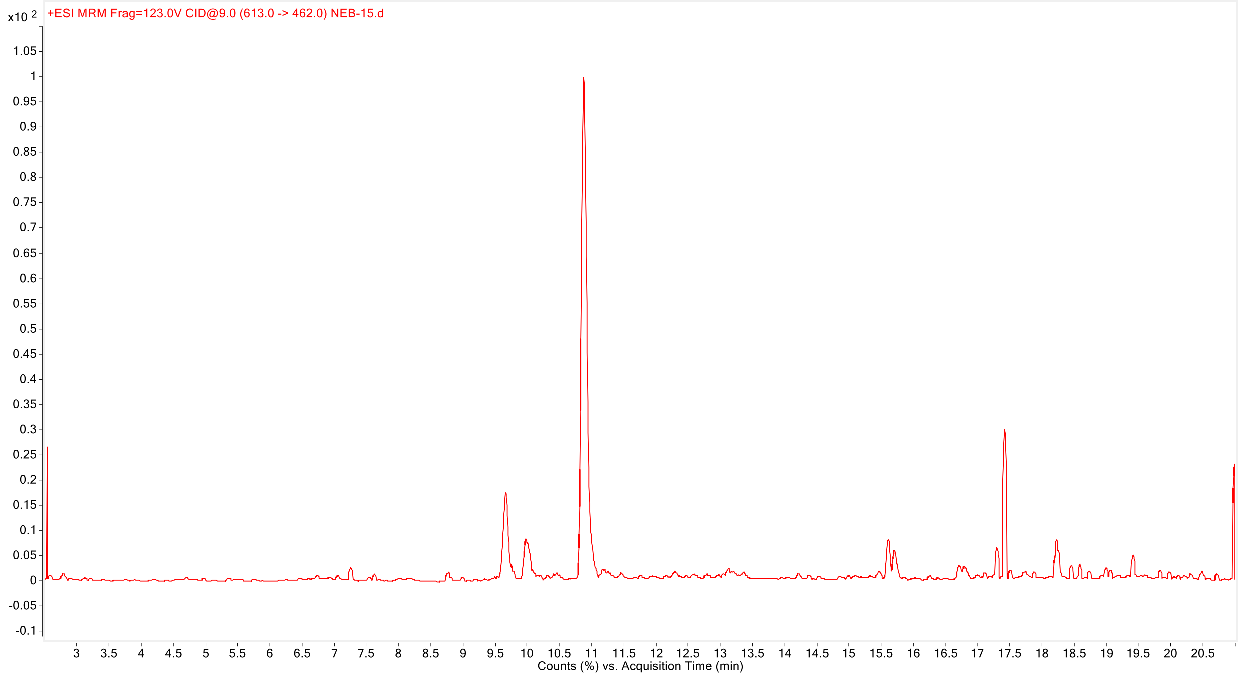

Suppl. Fig. S3. ScoMcrA digestion of pBR322 (Dcm$^+$ or Dcm$^-$) in Mn^{2+} buffer. A. Dcm$^+$ pBR322. **B.** Dcm$^-$ pBR322. The cleavage consensus sequence derived from multiple cleavage sites (WebLogo). **C.** cleavage sites in Dcm$^+$ pBR322. **D.** cleavage sites in Dcm$^-$ pBR322. Down arrows (↓)indicate the nicked strand. Up arrows (↑) indicate the bottom-strand nicking. Dcm MTase modifies the CCWGG to become C5mCW<u>G</u>G where the cytosine opposite to <u>G</u> is also symmetrically methylated. Doublets indicate the cleavage positions. The cut site determination and run-off sequencing follow the published procedure (2).

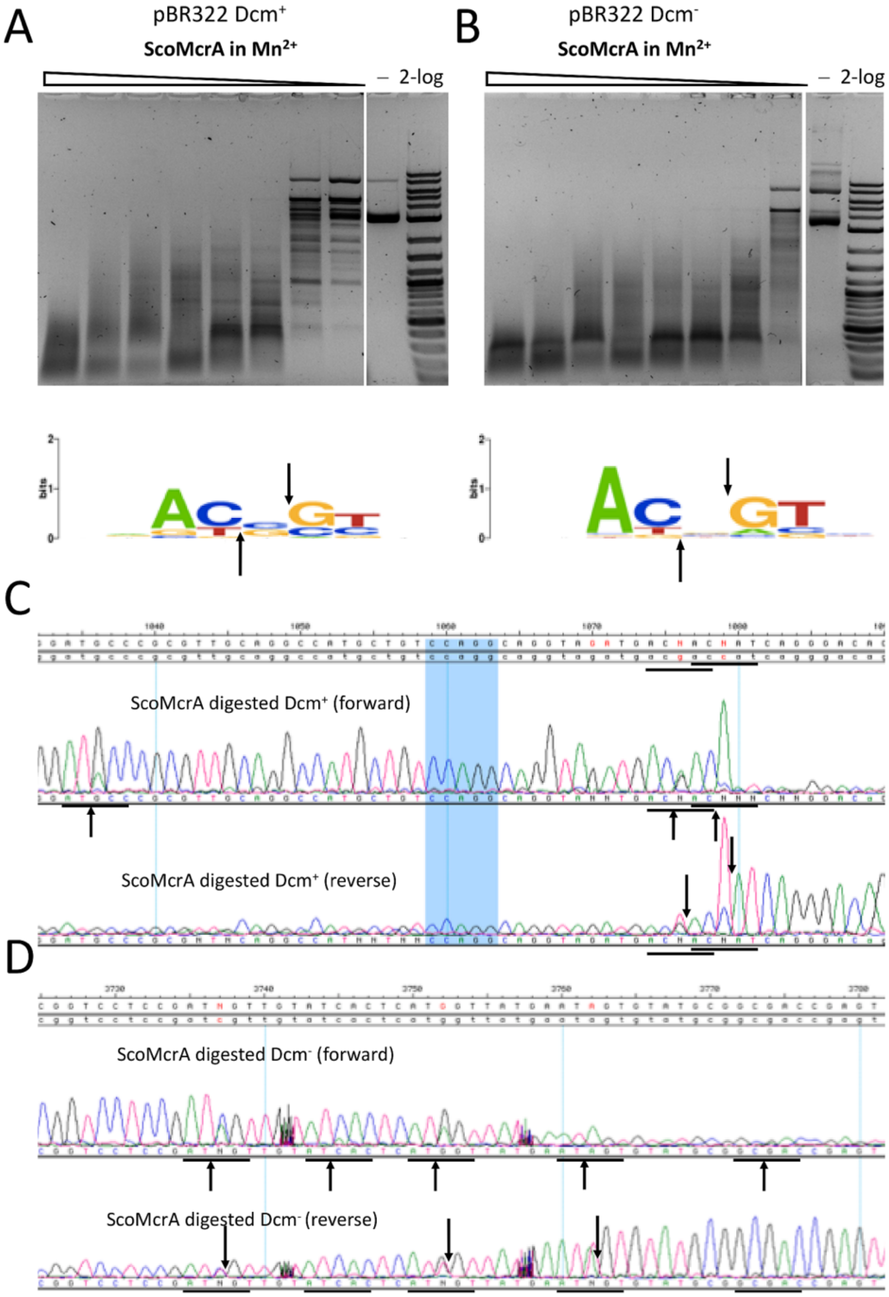

Suppl. Fig. S4. SDS-PAGE analysis of partially purified PT-dependent REases. A. SprMcrA-S (chitin column, predicted MW 25.6 kDa) and SprMcrA (chitin and heparin column, predicted MW 35.3 kDa). SpMcrA-S (short) is an N-terminally truncated version of SprMcrA. **B.** EcoWI (chitin and heparin column). FT, flow-through fractions. The predicted molecular mass of EcoWI is ~33.3 kDa. However, it appears as a 30 kDa protein in the SDS-PAG gel. **C.** Ksp11411 and Bsp305I following chitin column purification and DEAE beads binding and washing in high salt; Bsp48385I, Mae9806I, Hba180I, and Sau43800I (chitin column purification/DTT cleavage). The predicted protein molecular masses are: Ksp11411I (33.6 kDa), Bsp305I (37.1 kDa), Bsp48385I (33.2 kDa), Mae9806I (37.4 kDa), Hba180I (34.3 kDa), and Sau43800I (31.6 kDa), respectively. The "?" mark indicates a weak protein band for Bsp48385I (this enzyme expressed poorly, possibly owing to non-specific endonuclease activity). M. protein size marker (10 to 200 kDa, NEB).

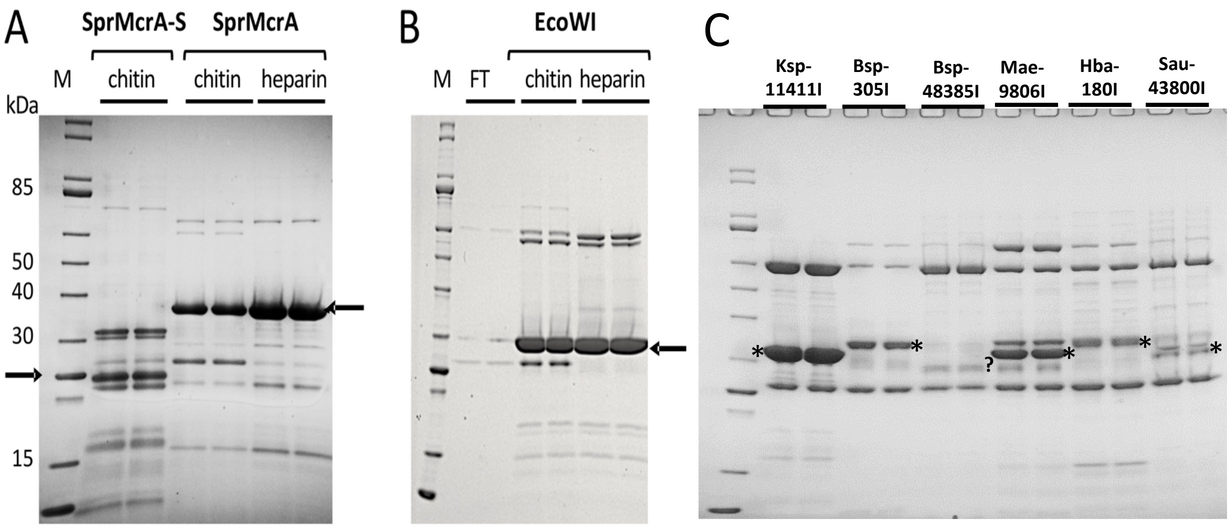

Suppl. Fig. S5. DNA sequencing to determine the nicking sites of SprMcrA-S (short) and consensus sequence derived from multiple nicking sites. A. DNA sequencing to detect the nicking sites. Uncut pBR322 was sequenced as a control. **B**. consensus sequence (SS↓RT) derived from multiple nicking sites compiled by WebLogo. The frequent nicking sites are similar to some phage encoded HNH nicking enzymes involved in phage *cos* site nicking and DNA packaging (3,4).

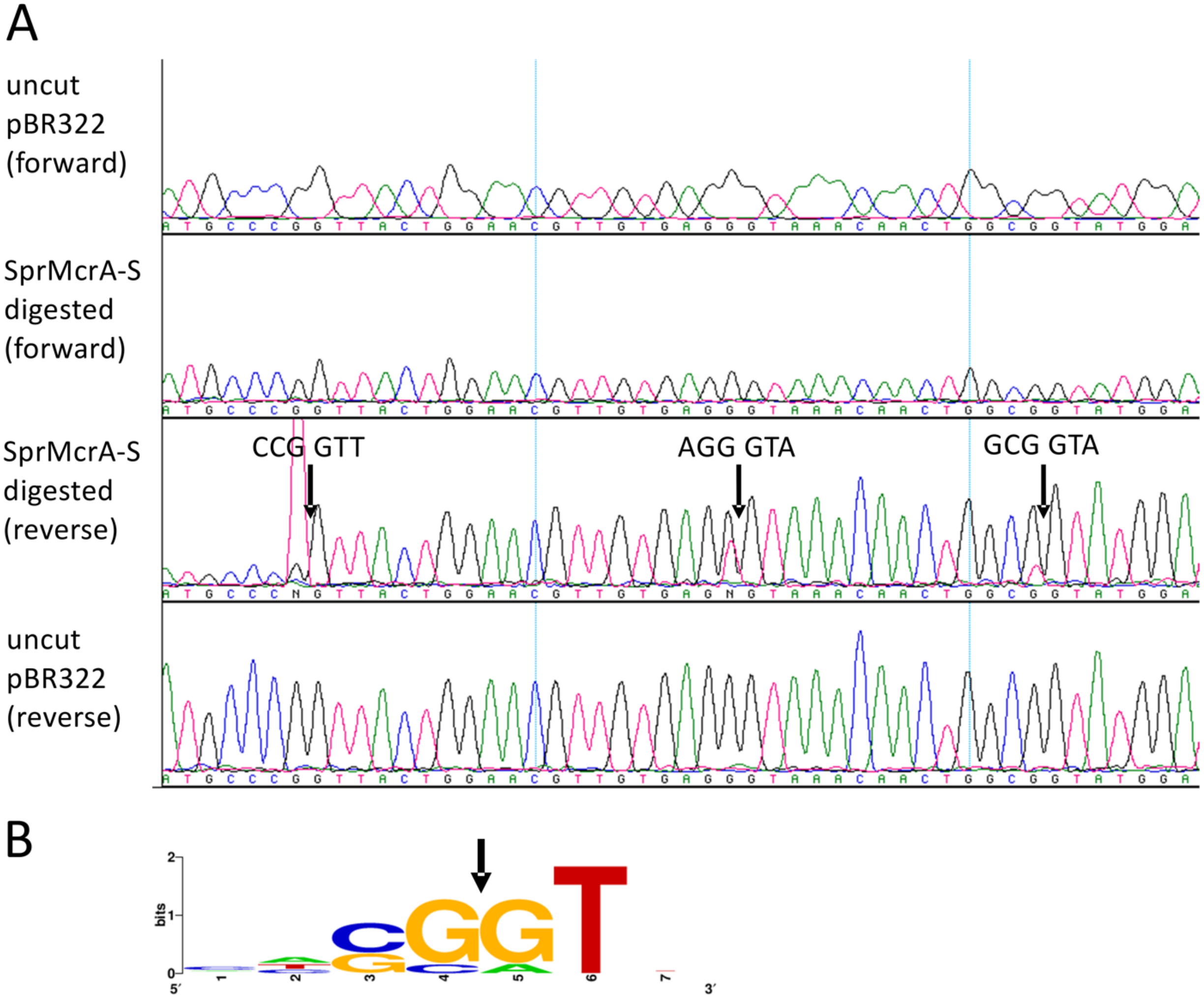

Suppl. Fig. S6. Cut site determination for EcoWI endonuclease. Dnd+ (SenC87) plasmid partially digested by EcoWI was subjected to run-off sequencing to determine the cleavage sites near GpsTTC modification site. Down arrows indicate the cleavage position in the strand shown. Up arrows indicate the nick in the bottom strand. EcoWI cleaves outside of its recognition sequence: GpsTTC N7/N6. Overlapping double peaks (doublet) indicate where cleavage takes place. The partial digestion was likely resulted from incomplete PT modifications at the particular site.

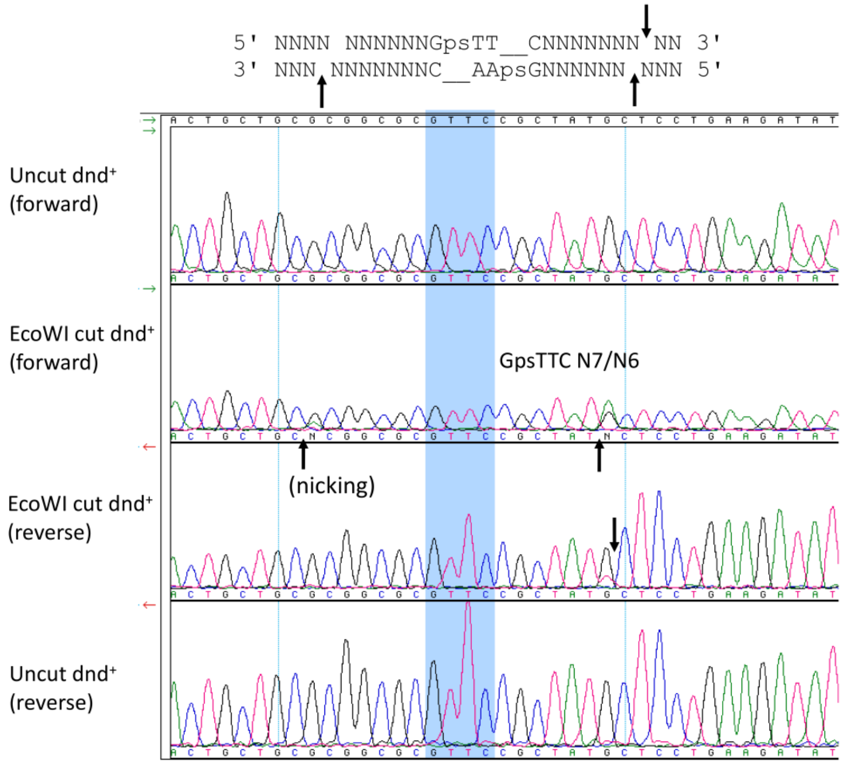

Suppl. Fig. S7. EcoWI digest of PT-modified oligos. Schematic diagram of duplex oligos and the possible cleavage products (top). Capillary electrophoresis (CE) of EcoWI-digested FAM-labeled oligos (bottom). The ~56 bp fragment in CE gels corresponds to the full length oligos. Shaded bars represent dsDNA.

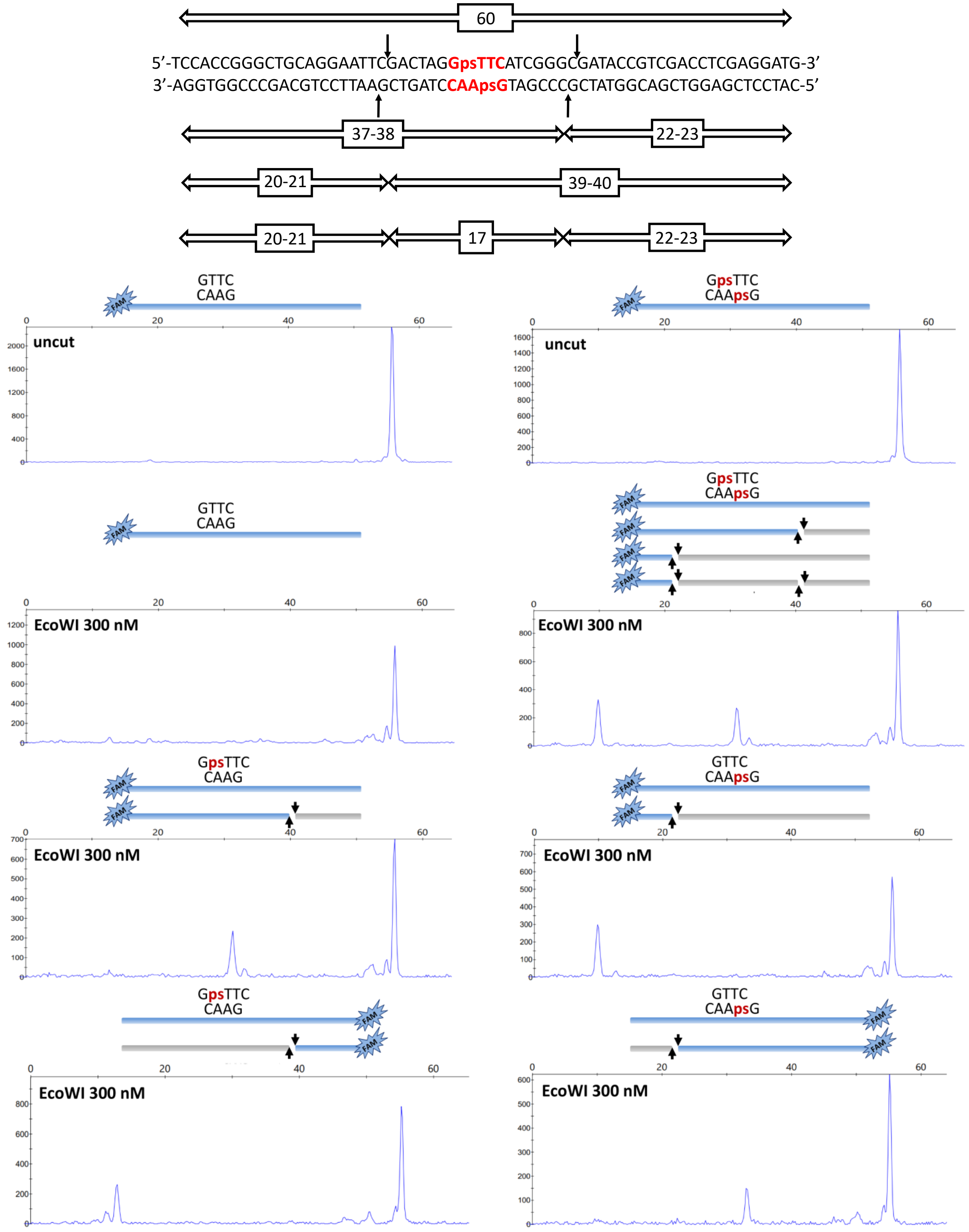

Suppl. Fig. S8. Analytical gel filtration chromatography of EcoWI endonuclease. A Superdex 200 10/300 GL column (24 ml bed volume; GE Healthcare) was run at 4°C at a flow of 0.38 ml/min. The partition coefficient (K_{av}) of the standard protein carbonic anhydrase (29 kDa), ovalbumin (43 kDa), and conalbumin (75 kDa) is plotted against log(mw) (filled diamonds). The K_{av} value of EcoWI (marked by x) was obtained from three independent runs under the same running conditions. EcoWI molecular weight of ~54.4 kDa was obtained from the standard curve (the insert equation).

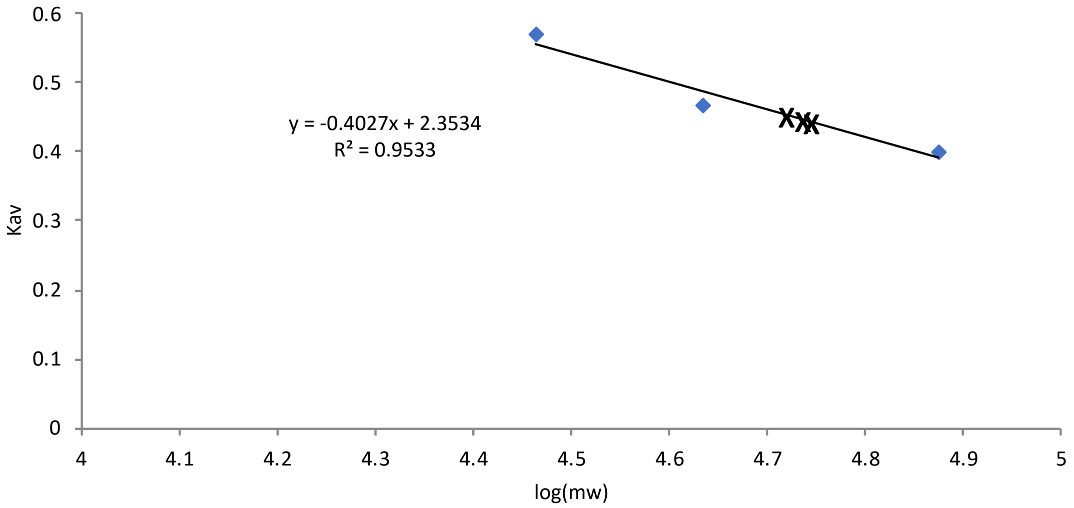

Ve	Vt	Kav	log(MW)	MW
15.27	23.56	0.4540	4.72	52.1
15.13	23.56	0.4448	4.74	54.9
15.07	23.56	0.4409	4.75	56.2

Suppl. Fig. S9. Ksp11411I activity assay on pBR322 and dnd+ (SenC87) plasmid in 1 mM Mn^{2+} buffer. The plasmids were digested in a medium salt buffer supplemented with 1 mM $MnCl_2$. The relative cleavage activity is at least 4-fold higher on dnd+ modified plasmid.

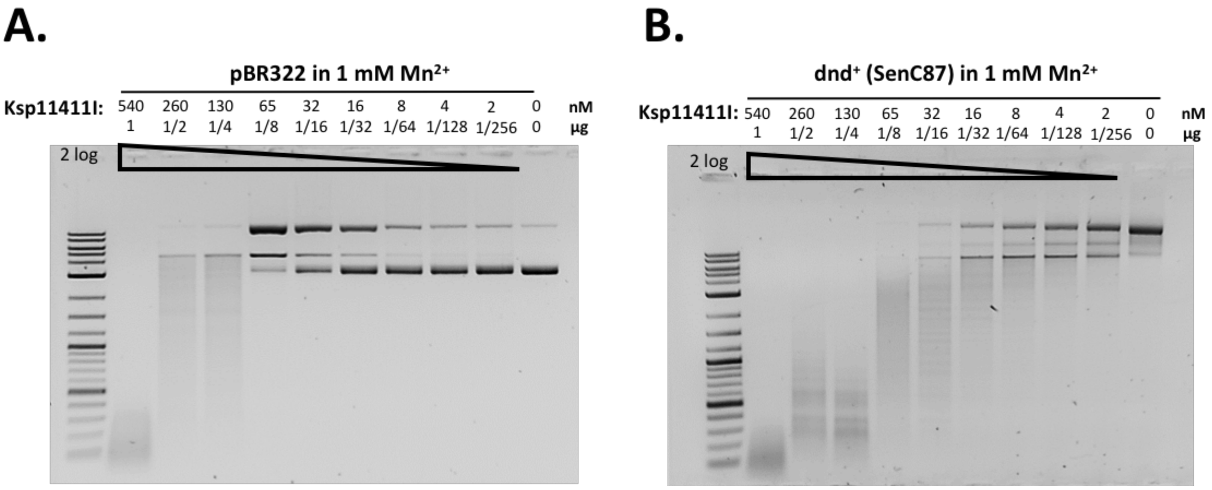

Suppl. Fig. S10. DNA sequencing of Ksp11411I digested plasmid. Left panel, dsDNA cleavage at GpsAAC N5/N4. Right panel, ssDNA nicks at GpsAAC N5 or GpsTTC N5.

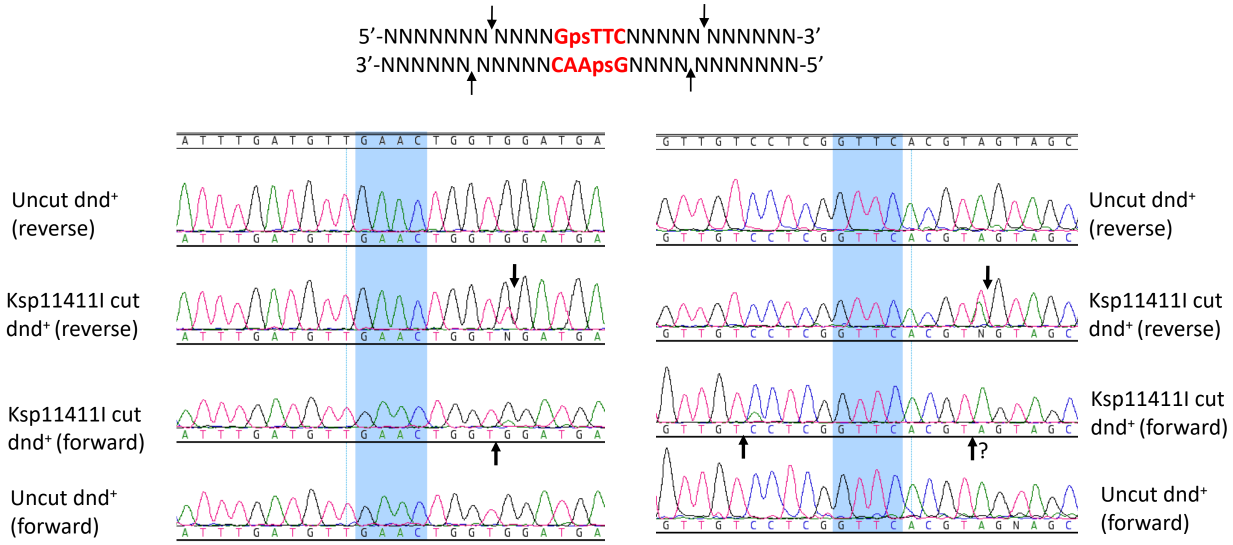

Suppl. Fig. S11. Ksp11411I activity assay on PT-modified oligos. See Suppl. Table S1 for the oligo sequences. Ksp11411I activity assay was carried out in a medium salt buffer with 0.1 mM Ni^{2+} at 37 °C for 60 min. It is also active in 1 mM Ni^{2+} buffer. Ksp11411I prefers to cleave hemi-PT-modified oligo (GpsTTC/GAAC) upstream of the GpsT dinucleotide, and thus differing from EcoWI which cleaves downstream of the hemi-PT modified oligos.

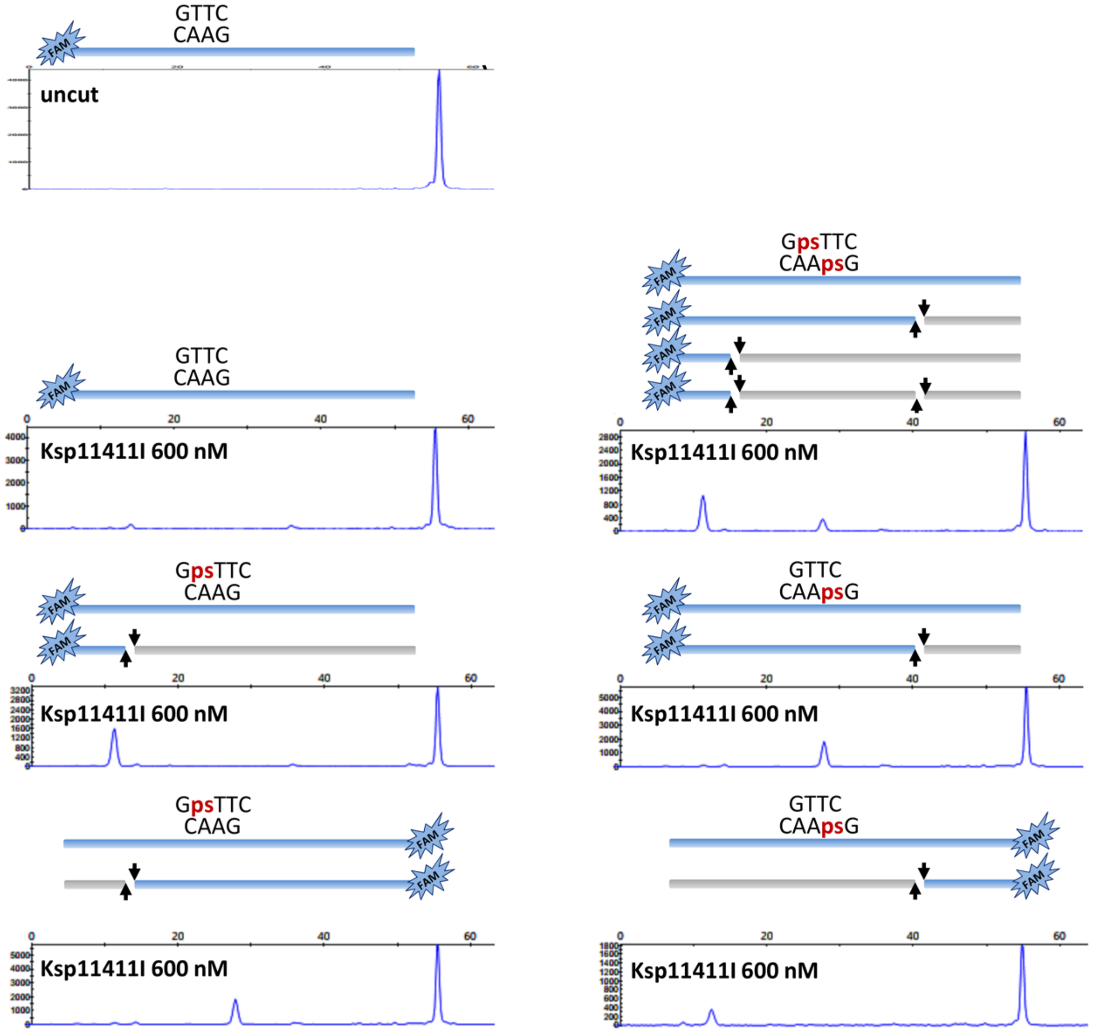

Suppl. Fig. S12. DNA sequencing of Bsp305I digested dnd^{+} plasmid (SenC87). Dnd^{+} plasmid was digested by Bsp305I in NEB buffer 2.1 (10 mM Mg^{2+}). Two cut sites are shown: GpsTTC N6/N5 and GpsAAC N6/N4 (the shorter N4 may result from exonuclease contamination or wobbled cleavage). It is known that certain REases may carry exonuclease activity in addition to endonuclease activity or imprecise wobble cut (5).

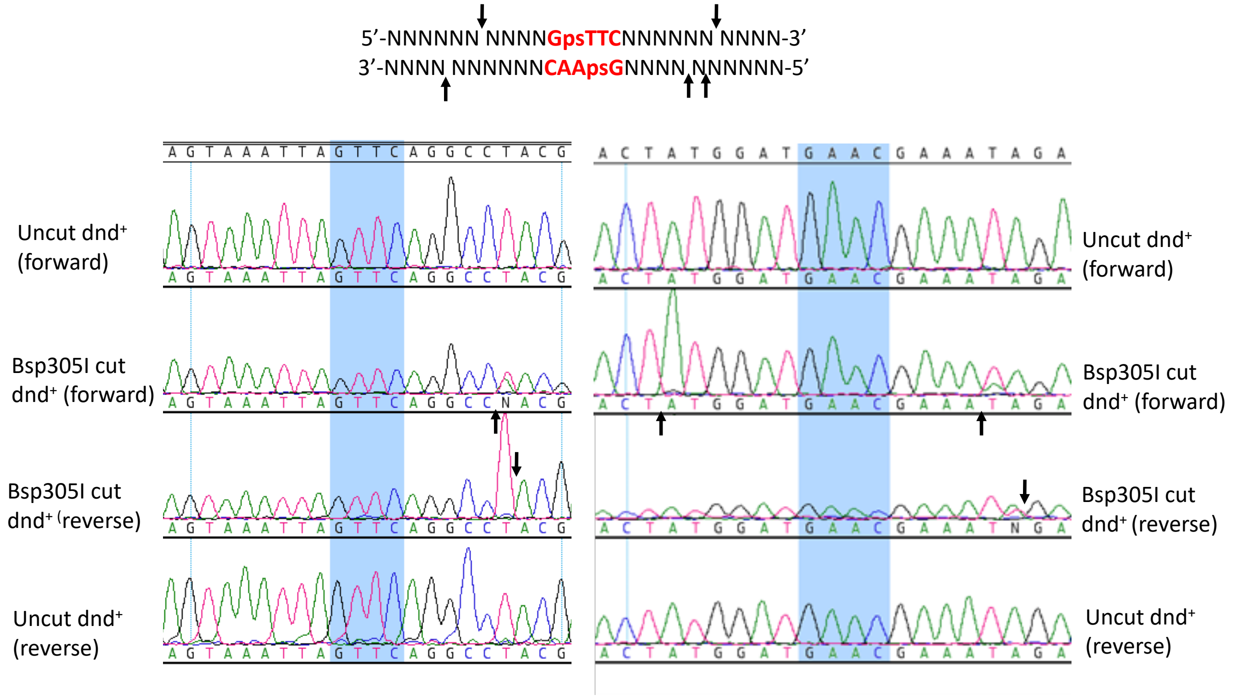

Suppl. Figure S13. CE assay for Bsp305I activity on PT-modified oligos in Mg^{2+} buffer. Bsp305I cleaved fully PT modified oligos only (under limited digestion condition). Hemi-modified oligos were poor substrates. FAM-labelled PT-modified or unmodified duplex oligos were shown on top of each peak scan.

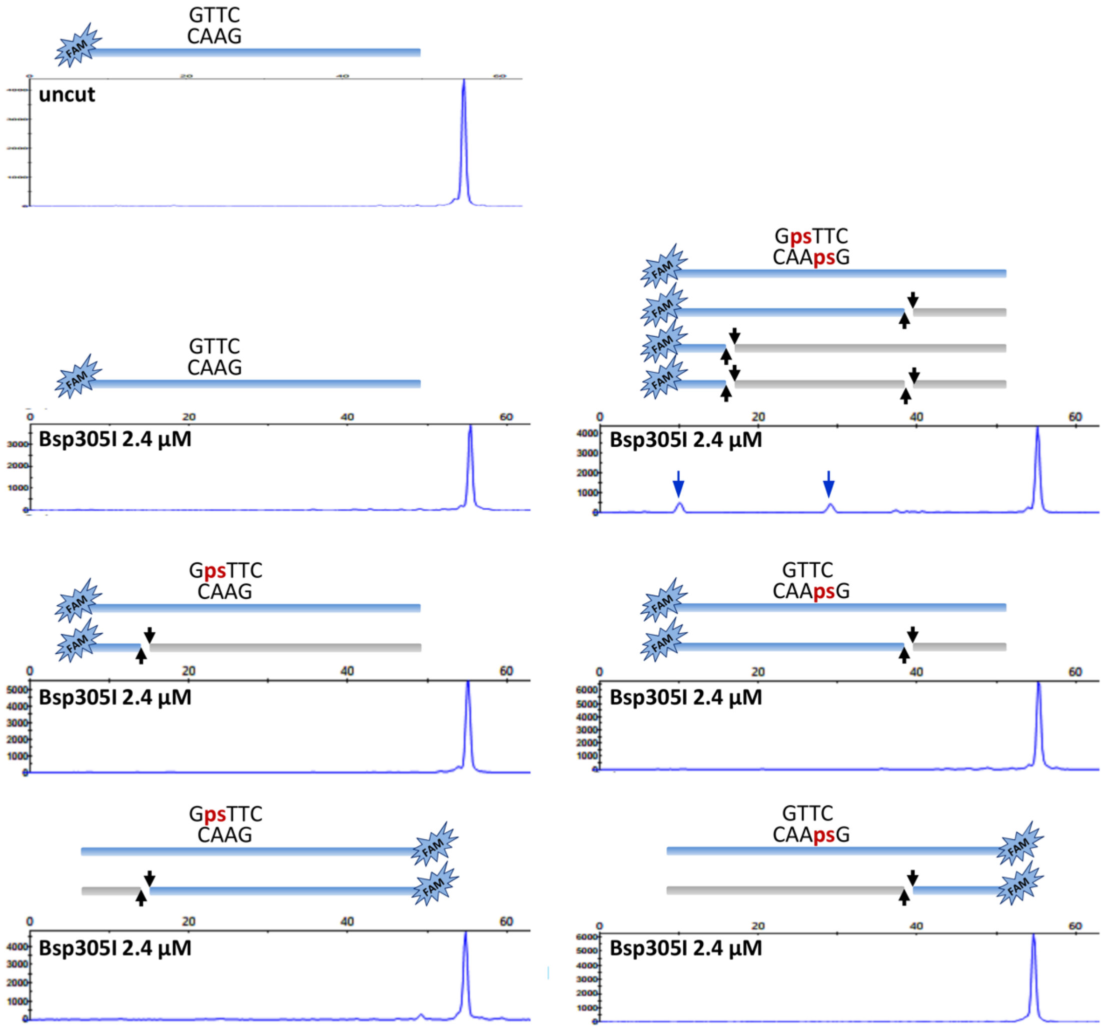

Suppl. Fig. S14. Bsp48385I mediated digestion of control and PT-modified plasmid and PCR DNA. A. pBR322 Dcm^{+} dnd^{-} and two dnd^{+} plasmids were subjected to Bsp48385I digestion in Mn^{2+} buffer. The enzyme partially digested plasmid DNA <u>regardless of PT modification</u>. **B.** The specificity of Bsp48385I was tested with control and PT-modified (with α-S-dATP incorporation in dNTP mix) 2.9 kb PCR DNA. Two major products were found in Bsp48385I digested unmodified PCR DNA (0.6 and 2.3 kb). However, Bsp48385I digested PT-PCR DNA to a range of smaller products (0.6 to 2.3 kb).

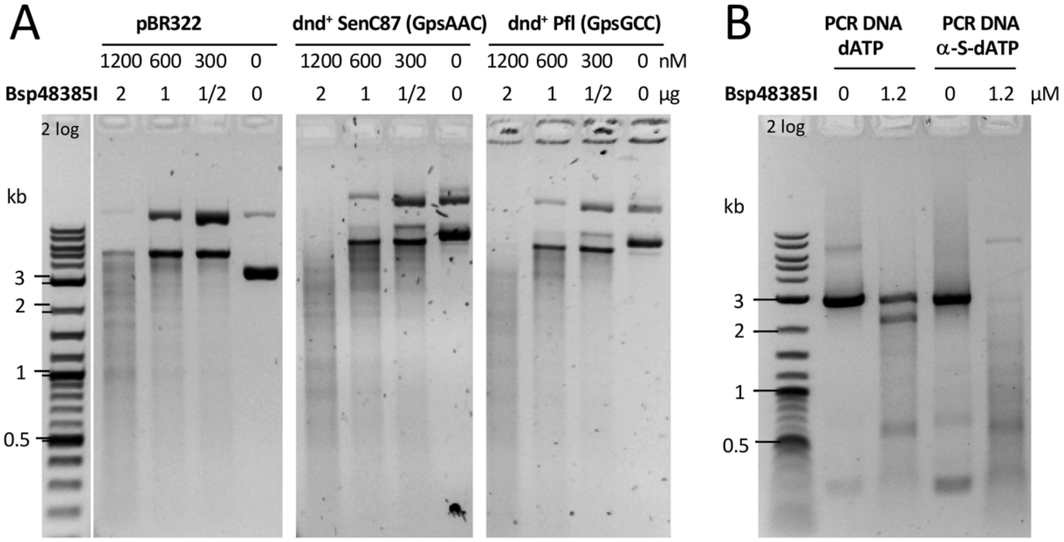

Suppl. Fig. S15. Mae9806I digestion of dnd^{-} and dnd^{+} plasmid DNA. Mae9806I cleavage of pBR322 and dnd^{+} plasmid was performed in Mn^{2+} buffer. Mae9806I shows approximately 4-fold preference for PT-modified DNA. Mae9806I displays low specific activity.

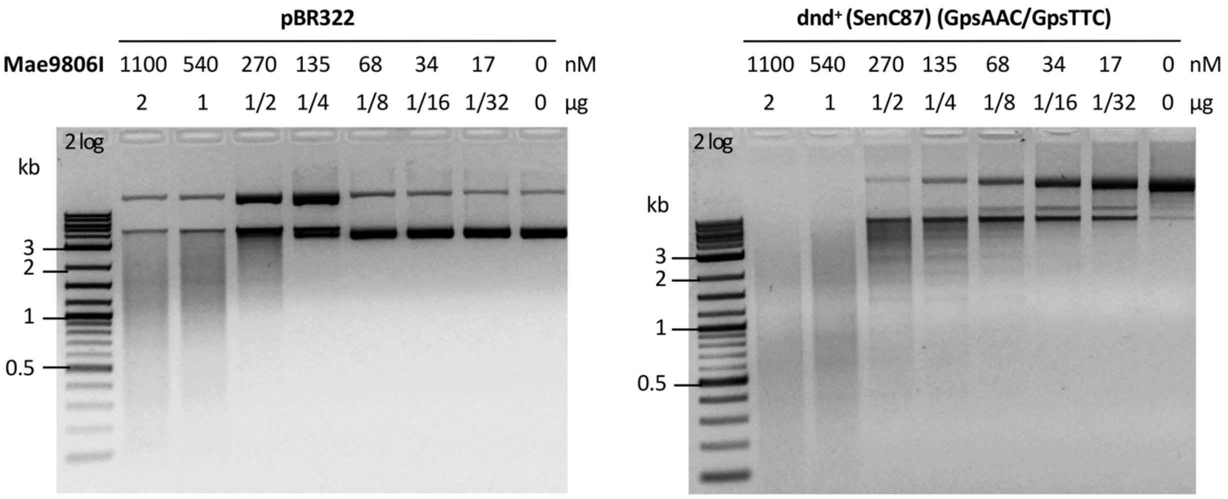

Suppl. Fig. S16. Mae9806I cut/nicked site determination by DNA sequencing. Dnd^{+} (SenC87) plasmid partially digested with Mae9806I was sequenced to detect single- and double-strand breaks near GpsAAC (GpsTTC) modified sites. The distance between the modification and cleavage site was variable.

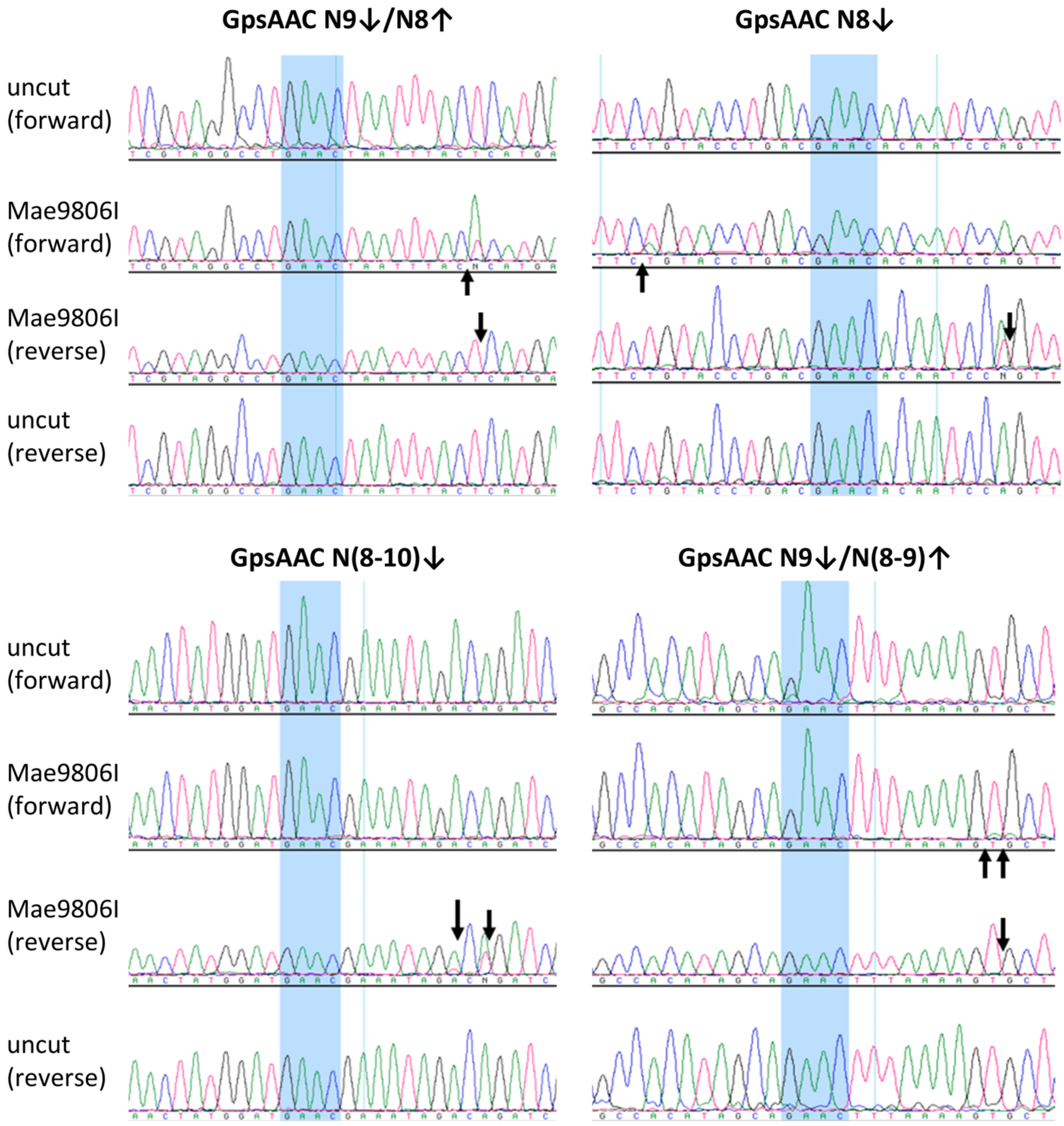

Suppl. Fig. S17. Sau43800I restriction activity assay in Mn^{2+} buffer and cut/nick site determination by DNA sequencing. A. Sau43800I digestion of pBR322 or dnd^{+} (SenC87) plasmid at 37ºC for 2 h. **B.** DNA sequencing of Sau43800I digested dnd^{+} plasmid (SenC87) to determine the cut site location (GTTC N8-9/N7-8). The shorter cleavage distance (N4) may be result of exonuclease contamination.

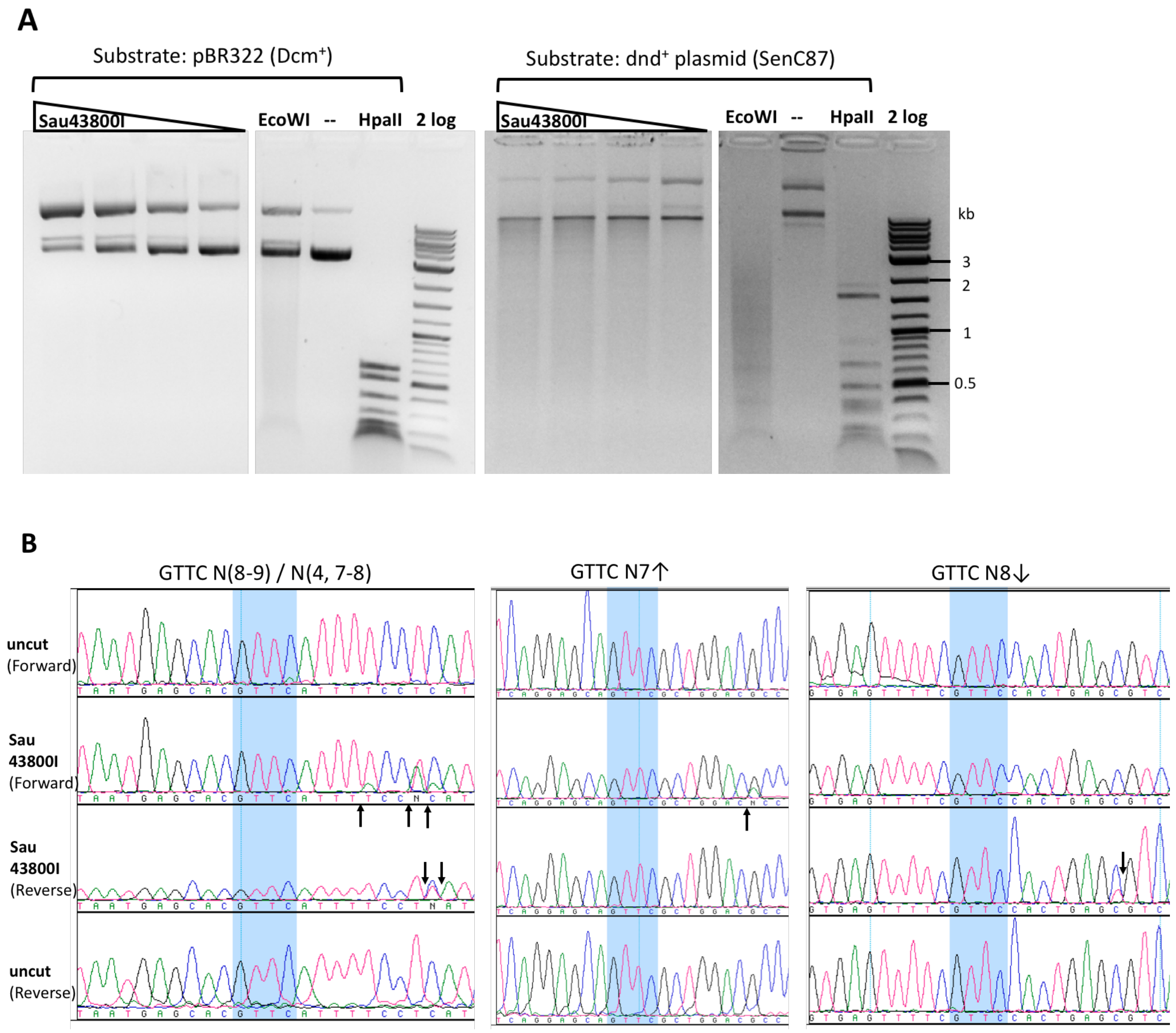

Suppl. Fig. S18. Hba180I restriction assay on dnd$^+$ plasmids and PT-modified PCR DNA substrates. **A.** Hba180I digestion of dnd$^+$ plasmids in Mn^{2+} buffer at 37ºC for 2 h (due to its low activity, longer digestion was required). Low activity (smearing) was detected on dnd$^+$ plasmid (SenC87). Low nicking activity was also detected on dnd$^+$ plasmid (Pfl) and pBR322 under the same condition. **B.** Hba180I digestion of PCR DNA (unmodified or PT modified). Although Hba180I displays non-specific activity on unmodified PCR DNA in Mn^{2+} buffer, PT modification in the PCR fragments appeared to stimulate the endonuclease activity. Most of the 2.9 kb fragment was degraded into smaller products. The cleavage sites were not determined due to its low endonuclease activity.

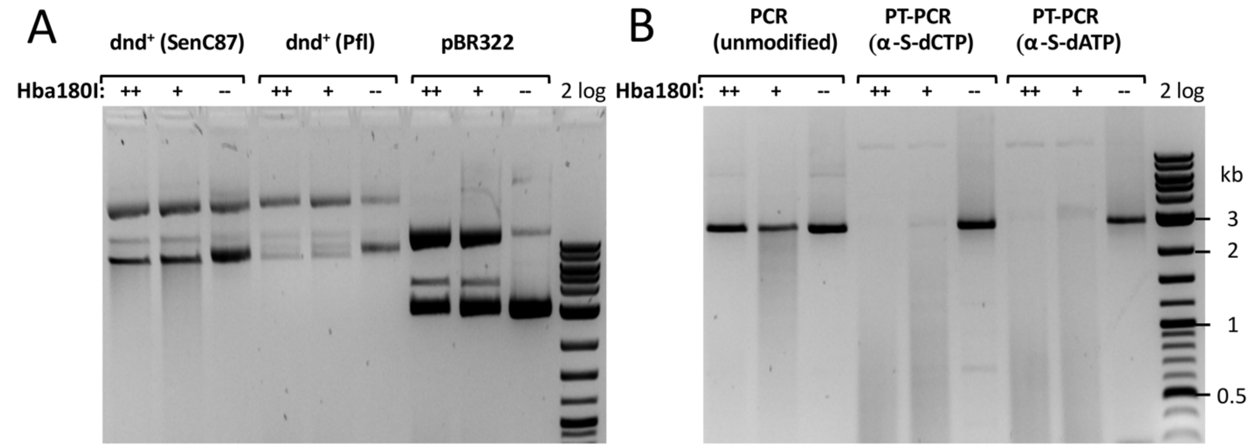

Suppl. Fig. S19. Multiple amino acid (aa) sequence alignment of EcoWI homologs by PROMALS3D. SBD, sulfur binding domain (predicted TRD). Zinc fingers (zinc binding sites): CxxC and CxxH. The variable linker region is shown by a bracket [---]. Arrows indicate the position of the predicted catalytic residues His, Asn (or Arg), and His (HNH). Low activity homologs are not included here. Predicted α helix (h) and β sheet (e) are shown below the aa sequence. The numbers (5-9) on top of each position indicate the level of conservation. The predicted P-L-W residues for PT specificity is partly missing in SprMcrA-S.

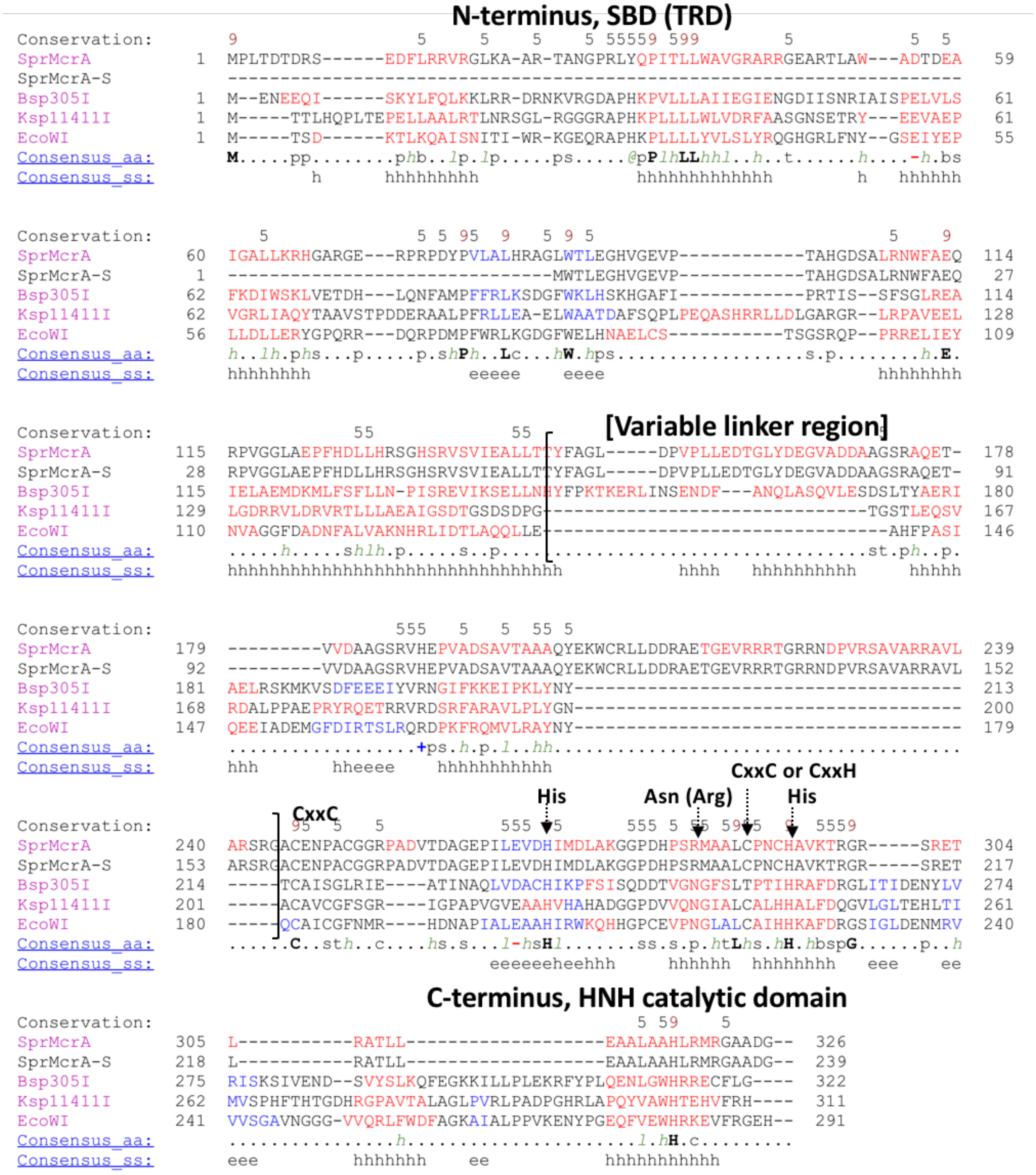

Suppl. Fig. 20. Sulfur binding domain (SBD) aa sequence alignment. **A.** Structure of ScoMcrA SBD in complex with PT-modified duplex oligo (PDB code: 5ZMO). The important aa residues H, R, Y, and P for PT backbone interactions are highlighted. **B.** Sequence alignment of the SBD domains of the enzymes that were subject of the current study. The secondary structure elements of the domain in the structure and above the alignment were colored in rainbow mode from N- to C-terminus (both were based on the ScoMcrA crystal structure). The alignment was created with the ClustalX program (http://www.clustal.org)(6).

A

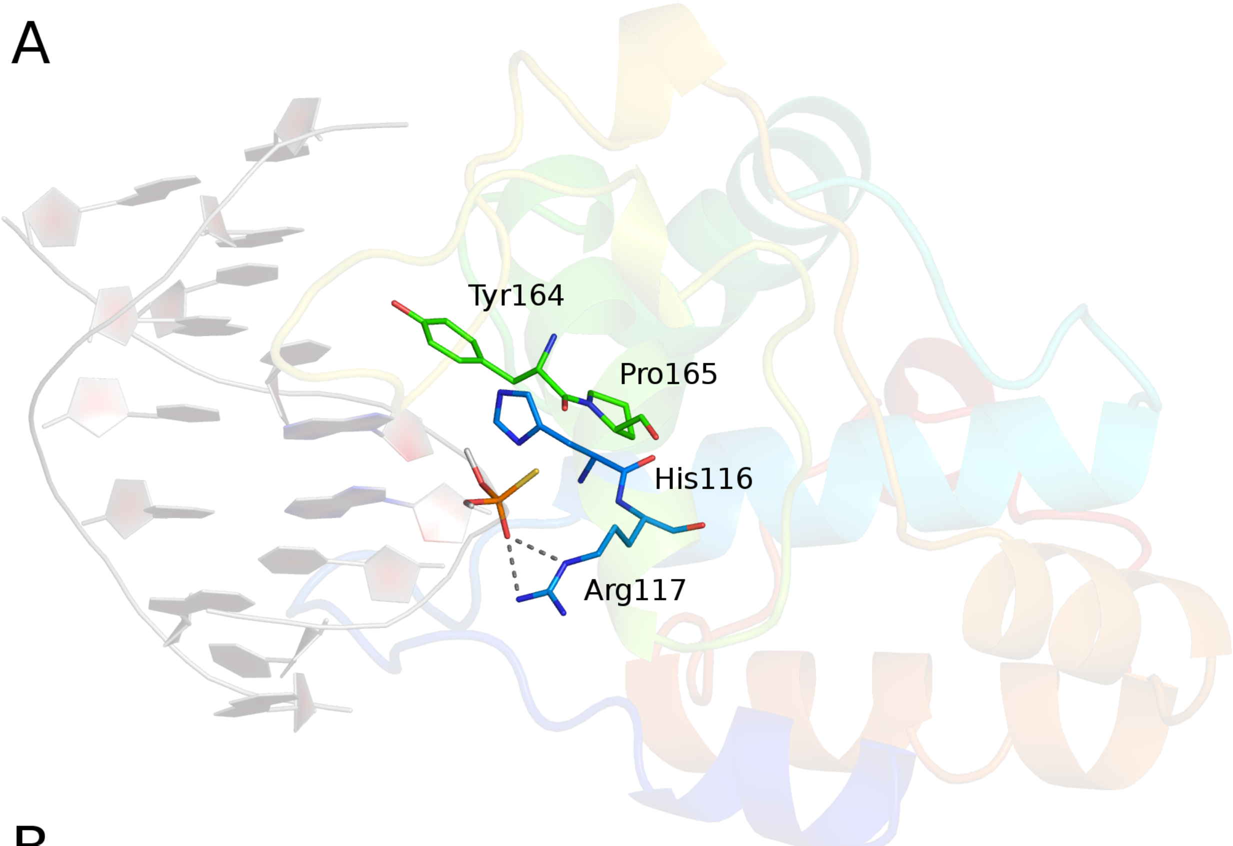

B

```
ScoMcrA      ---------PKTFHRRVGDVRPARR-AMGPALHRPVLLLWAIGQAVARAPRLQPWS---TTRDAVAPLMEKYGQ--VEDG
SprMcrA      -MPLTDTDRSEDFLRRVRGLKAART-ANGPRLYQPITLLWAVGRARRGEARTLAWA---DTDEAIGALLKRHGA---RGE
EcoWI        ------MTSDKTLKQAISNITIWRK-GEQRAPHKPLLLLYVLSLYRQGHGRLFNYGS--EIYEPLLDLLERYGP---QRR
Ksp11411     MTTLHQPLTEPELLAALRTLNRSGLRGGGRAPHKPLLLLWLVDRFAASGNSETRYE---EVAEPVGRLIAQYTAAVSTPD
Bsp305I      ---MENEEQISKYLFQLKKLRRDRNKVRGDAPHKPVLLLAIIEGIENGDIISNRIAI---SPELVLSFKDIWSKLVETDH
Mae9806I     MVDSLPLRNLKYYCDLFSKIKVNKSKQSGEAPYKPILLLSVIELISDSQIQDNKIYISDELIDTFDKYWKRLNP---DAF
Bsp58385     -------MEINKLIEELDKIRTYKGSKIGASIKKPLFLLLLISLIEGGKVKRNKFYFP-DIERELDLLIRTFGG---RVG
Sau43800I    -------MSEEEVLSRLSSLRQHRW-QGRRSPHKPLLVLLALGRLTTTGTSEVPWS----MAGKLAELIRDFGPPSATQP
Hba180I      --------MIENFYKWYSNLNKNKV-QQRYSPHKPLTIIYTLAKSLKG-IRWIEYN---KDRVLLEDLIAEHTN---FKT

ScoMcrA      VDGVRYPFWALVRDD----LWCVEQAEELTLTSRGRRPTLESLNAVDPSAGLREDDYNLLRSQPEAAASAAAGLIARYFH
SprMcrA      RPRPDYPVLALHRAG----LWTLEGHVGEVPTAHGDSALRNWFAEQRPVGGLAEPFHDLLHRSGHSRVSVIEALL-----
EcoWI        DQRPDMPFWRLKGDG----FWELHNAELCSTSG-SRQPPRRELIEYNVAGGFDADNFALVAKNHRLIDTLAQQLLE----
Ksp11411     DERAALPFRLLEAE-----LWAATDAFSQPLP---EQASHRRLLDLGARGRLRPAVEELLGD---------RRVLDRVRT
Bsp305I      LQNFAMPFFRLKSDG----FWKLHSKHGAFIPR--TISSFSGLREAIELAEMDKMLFSFLLN------PISREVIKSELL
Mae9806I     KGGLALPFFHLKNDG----FWKLKFSDSYDGGR---PQTIPKIRADVDYAILDEELYKLLRE-PEARRQLIDTLIEAWFS
Bsp58385     KGKPEQPFHHLNSSI----IWDIHVPYGTRFSN-KETLPISVLRDPETYGYINNEIFKLLSSDNIARAEAAQFILGKYWP
Sau43800I    AQSAAYPFTRLRSDG----IWVLD----HDVPM-DRVQP---LAAHGVVGRLAPP-VEDALRDAGIAAAAARRLVESEFP
Hba180I      RPNCLHPIWRLKNDSKEIDLWQVIPTT-LNVNQ-SGDISQSEAKSINLKAGFSDRFYNWLQEHKAEGKELIKFVISDNFP
```

Suppl. Fig. S21. Gene neighborhood analysis and ORFs next to the PT-dependent REases (PTDRs). Only the immediate ORFs adjacent to the PTDRs are shown. ORFs were predicted by aa sequence similarity to known proteins by BlastP. Only Bsp48385I is associated with Dnd^+ modification gene cluster *dndBCDEA* and the restriction cluster *dndFGH*. One small ORF (*dndX*, 124 aa) possibly encoding a transcriptional regulator is located between *dndH* and *dndG*. The complex is presumably formed by DndA-E and DndFGH to restrict unmodified DNA. ORFs are annotated in REBASE (7).

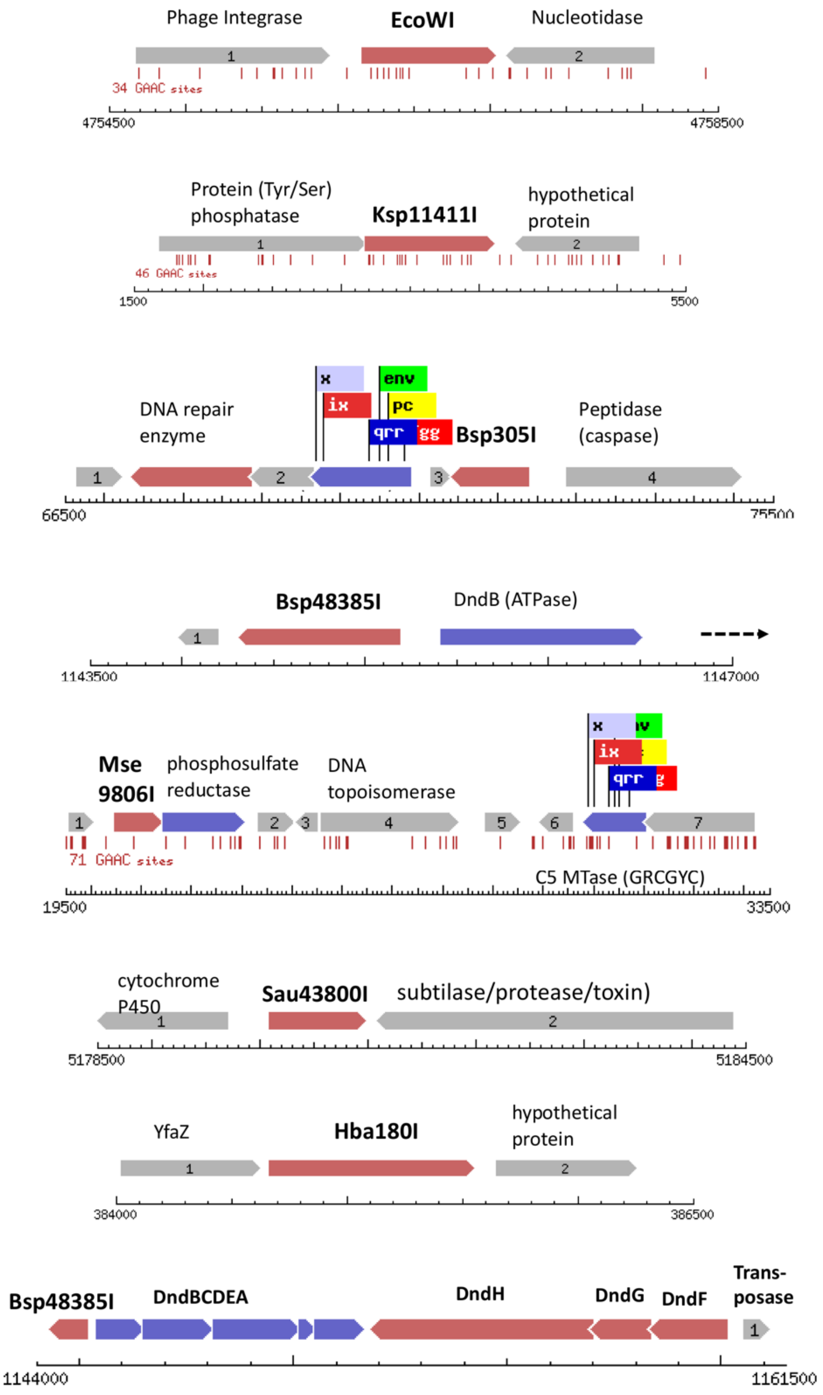

References

1. Wang, L., Chen, S., Vergin, K.L., Giovannoni, S.J., Chan, S.W., DeMott, M.S., Taghizadeh, K., Cordero, O.X., Cutler, M., Timberlake, S. *et al.* (2011) DNA phosphorothioation is widespread and quantized in bacterial genomes. *Proc Natl Acad Sci U S A*, **108**, 2963-2968.
2. Lutz, T., Flodman, K., Copelas, A., Czapinska, H., Mabuchi, M., Fomenkov, A., He, X., Bochtler, M. and Xu, S.Y. (2019) A protein architecture guided screen for modification dependent restriction endonucleases. *Nucleic Acids Res*, **47**, 9761-9776.
3. Xu, S.Y. and Gupta, Y.K. (2013) Natural zinc ribbon HNH endonucleases and engineered zinc finger nicking endonuclease. *Nucleic acids research*, **41**, 378-390.
4. Kala, S., Cumby, N., Sadowski, P.D., Hyder, B.Z., Kanelis, V., Davidson, A.R. and Maxwell, K.L. (2014) HNH proteins are a widespread component of phage DNA packaging machines. *Proc Natl Acad Sci U S A*, **111**, 6022-6027.
5. Yu, H., Liu, G., Zhao, G., Hu, W., Wu, G., Deng, Z. and He, X. (2018) Identification of a conserved DNA sulfur recognition domain by characterizing the phosphorothioate-specific endonuclease SprMcrA from *Streptomyces pristinaespiralis*. *Mol Microbiol*, **110**, 484-497.
6. Thompson, J.D., Gibson, T.J. and Higgins, D.G. (2002) Multiple sequence alignment using ClustalW and ClustalX. *Curr Protoc Bioinformatics*, **Chapter 2**, Unit 2 3.
7. Roberts, R.J., Vincze, T., Posfai, J. and Macelis, D. (2010) REBASE--a database for DNA restriction and modification: enzymes, genes and genomes. *Nucleic Acids Res*, **38**, D234-236.
